# Effects of Agricultural Pesticides in Aquafeeds on Wild Fish Feeding on Leftover Pellets Near Fish Farms

**DOI:** 10.3389/fgene.2019.00794

**Published:** 2019-09-26

**Authors:** Pål A. Olsvik, Anett Kristin Larsen, Marc H. G. Berntssen, Anders Goksøyr, Odd André Karlsen, Fekadu Yadetie, Monica Sanden, Torstein Kristensen

**Affiliations:** ^1^Institute of Marine Research (IMR), Bergen, Norway; ^2^Faculty of Biosciences and Aquaculture, Nord University, Bodø, Norway; ^3^Department of Research and Development, UiT – The Arctic University of Norway, Tromsø, Norway; ^4^Department of Medical Biology, UiT – The Arctic University of Norway, Tromsø, Norway; ^5^Department of Biological Sciences, University of Bergen, Bergen, Norway

**Keywords:** aquaculture, fish feed, insecticides, chlorpyrifos-methyl, wild fish exposure, metabolomics, transcriptomics

## Abstract

Screening has revealed that modern-day feeds used in Atlantic salmon aquaculture might contain trace amounts of agricultural pesticides. To reach slaughter size, salmon are produced in open net pens in the sea. Uneaten feed pellets and undigested feces deposited beneath the net pens represent a source of contamination for marine organisms. To examine the impacts of long-term and continuous dietary exposure to an organophosphorus pesticide found in Atlantic salmon feed, we fed juvenile Atlantic cod (*Gadus morhua*), an abundant species around North Atlantic fish farms, three concentrations (0.5, 4.2, and 23.2 mg/kg) of chlorpyrifos-methyl (CPM) for 30 days. Endpoints included liver and bile bioaccumulation, liver transcriptomics and metabolomics, as well as plasma cholinesterase activity, cortisol, liver 7-ethoxyresor-ufin-O-deethylase activity, and hypoxia tolerance. The results show that Atlantic cod can accumulate relatively high levels of CPM in liver after continuous exposure, which is then metabolized and excreted via the bile. All three exposure concentrations lead to significant inhibition of plasma cholinesterase activity, the primary target of CPM. Transcriptomics profiling pointed to effects on cholesterol and steroid biosynthesis. Metabolite profiling revealed that CPM induced responses reflecting detoxification by glutathione-S-transferase, inhibition of monoacylglycerol lipase, potential inhibition of carboxylesterase, and increased demand for ATP, followed by secondary inflammatory responses. A gradual hypoxia challenge test showed that all groups of exposed fish were less tolerant to low oxygen saturation than the controls. In conclusion, this study suggests that wild fish continuously feeding on leftover pellets near fish farms over time may be vulnerable to organophosphorus pesticides.

## Introduction

Norway produces annually about 1.3 million metric tons of Atlantic salmon (*Salmo salar*) (Ytrestøyl et al., 2015). For each kilogram of salmon produced, about 0.5 kg of feces and uneaten feed pellets is generated (Svåsand et al., 2017). The majority of this waste will, depending on bottom topography and ocean currents, slip through the open-cage net pens and accumulate in sediments beneath the fish farms. Because the fish feed is nutritious and high in energy, wild organisms tend to feed on this organic waste. Uneaten feed pellets are typically eaten by wild fish that aggregate in large numbers around fish farms (Uglem et al., 2014). Numerous fish species have been observed underneath salmon farms, the most common being Atlantic cod (*Gadus morhua*), saithe (*Pollachius virens*), haddock (*Melanogrammus aeglefinus*), and Atlantic mackerel (*Scomber scomber*) (Dempster et al. 2009). Environmental hazards of this organic waste include spreading of pathogens from salmon farms to wild fish, spreading of orally administered drugs used against salmon lice, local eutrophication, and oxygen depletion, as well as contaminants in the feeds such as heavy metals and persistent organic pollutants.

Today, feed used in the seawater production phase of Atlantic salmon aquaculture typically contains 70% plant ingredients (Ytrestøyl et al., 2015). By replacing marine ingredients with plant material in the feed, the amount of feces generated per kilogram of produced salmon has increased (Aas et al., 2016). As a carnivorous species, salmon has a poor ability to digest some plant-based ingredients (Gatlin et al., 2007). Substitution of fish ingredients with plant ingredients in the feeds also introduces new unwanted substances. Some of the most commonly used pesticides in terrestrial agriculture have been detected in salmon feeds. For example, recent wide-scale screening of aquafeeds has shown that they might contain chlorpyrifos-methyl (CPM) (Nacher-Mestre et al., 2014; Portoles et al., 2017). In commercially available Atlantic salmon feeds surveilled in 2017, the levels of CPM reported ranged from 11 to 26 μg/ kg (Sele et al., 2018). In average, about 5–10% of surveilled feed samples contain CPM levels above the detection limit.

Chlorpyrifos (CPF) is a widely used agricultural organophosphorus pesticide (OP) (Dow Chemical Company, 2018) that can be highly toxic to fish (Schimmel et al., 1983; Yen et al., 2011). Inhibition of acetylcholinesterase in nerve cells is the main effect of OPs in fish (Yen et al., 2011). Observed secondary effects include oxidative stress, immunotoxicity, neurotoxicity, endocrine disruption, and gut microbiota dysbiosis (Levin et al., 2003; Eddins et al., 2010; Oruc, 2010; Sharbidre et al., 2011; Yen et al., 2011; Richendrfer et al., 2012; Xing et al., 2015; Zhang et al., 2017; Wang et al., 2019). In an effort to study the molecular effects of OPs found in salmon feeds, we have been conducting *in vitro* and *in vivo* exposure studies with CPF, pirimiphos (PP), and their methylated forms in Atlantic salmon (Olsvik et al., 2015; Olsvik et al., 2017; Sanden et al. 2018; Olsvik et al., 2019). These studies have shown that CPF and PP in particular disrupt lipid metabolism and induce oxidative stress in liver of exposed fish.

To our knowledge, no attempts have been made to document how OPs affect marine fish species living mainly on waste feed near fish farms. Although fish can metabolize and excrete OPs, continuous dietary exposure may lead to bioaccumulation and impacts on fish health. The aim of this study was therefore to search for novel biomarkers of CPM exposure in Atlantic cod, one of the most common fish species aggregating around Atlantic salmon farms in Norway, using a functional genomics approach with global transcriptomic and metabolomic profiling. Juvenile Atlantic cod with end weight of 223 ± 8 g was exposed to three doses of CPM (0.5, 4.2, or 23.2 mg/kg feed). Liver tissue was harvested after 30 days of dietary exposure and used for transcriptomic and metabolite analyses, as well as 7-ethoxyresorufin-O-deethylase (EROD) activity. Accumulation of CPM and its main metabolite 3,5,6-trichloro-2-pyridinol (TCP) was determined in liver and bile. Plasma was analyzed for cortisol levels, cholinesterase activity, aspartate aminotransferase (ASAT), alanine aminotransferase (ALAT), and total protein. A gradual hypoxia challenge test was included to search for possible physiological impairment and phenotypic effects of CPM exposure. In salmon, reduced hematocrit was observed after 30 days of dietary CPM exposure (Sanden et al., 2018). In this study, we therefore hypothesized that CPM might impair the fish performance in a dose-dependent manner when challenged by adverse environmental conditions such as hypoxia. None observed dose–response effect levels of dietary CPM exposure were assessed by lower benchmark dose (BMDL) models established by the European Food Safety Authorities (EFSA, 2017a; Berntssen et al., 2018). The BMDL assessment was performed on the dose–response effects on general adverse effect parameters (e.g., plasma biochemistry and growth) as well as specific biomarkers of CPM as assessed by both transcriptomic and metabolite responses (e.g., metabolic responses on lipids and gene expression). In addition to biological responses, a BMDL assessment was made on the formation of TCP in both liver and bile. A risk assessment on potential harmful effect on fish health when feeding on left-over pellets was made by comparing the BMDL response in fish and surveyed CPM levels in commercially produced Norwegian Atlantic salmon feeds.

## Materials and Methods

### Exposure Experiment

Locally bred Atlantic cod (150 ± 2 g, mean ± SEM, *n* = 276) were obtained and kept at the Tromsø Aquaculture Research Station, Kårvika, Tromsø, Norway. Prior to the acclimatization period, the fish were anesthetized with 0.07 g/l tricaine methanesulfonate (Scan Aqua AS, Årnes, Norway) and individually inserted with a single-load PIT-tag (Biomark, Biose, Idaho, USA). The fish were distributed among 12 fiberglass tanks (500 L, 23 fish per tank) supplied with filtered seawater in a triplicate tank design (three tanks per treatment). During a 2-week acclimatization period to holding facilities (May 7– 21), all fish were fed the control diet that contained no CPM. The exposure experiment lasted for 30 days (May 22–June 21). During this period, the fish received one of the four experimental diets: control with no CPM, low: 0.5, medium: 4.2, and high: 23.2 mg CPM/kg. The exposure concentrations were selected based on an earlier 67-day dietary exposure experiment with CPM and Atlantic salmon (Sanden et al., 2018; Olsvik et al., 2019). To determine the average daily dose of CPM consumed (nanogram per kilogram fish per day, **Table 1**), feed intake per tank was quantified by collecting feed waste once a day post-feeding. The fish were reared in seawater (salinity 34 g/L, 7°C) using a 24-h light, 0-h dark photoperiod regimen (natural photoperiod for 69°N). Fish were euthanized by an overdose of tricaine methanesulfonate followed by a quick blow to the head. After collection, plasma and liver samples were stored in liquid nitrogen before downstream analysis.

**Table 1:** Concentration of chlorpyrifos-methyl in the experimental diets (nominal values, *n* = 3), and estimated daily dose (feed given minus collected waste each day).

Nominal feed concentration	Measured feed concentration	Estimated daily dose of chlorpyrifos-methyl
mg/kg	mg/kg	µg/kg fish per day
		
Control	<0.01	<0.01
Low (0.5)	0.50	0.68 ± 0.24
Medium (5.0)	4.2	5.52 ± 0.98
High (25.0)	23.20	36.2 ± 10.1

Specific growth rate (SGR), feed intake and feed conversion (FC) rate were calculated with the following equations. SGR was assessed for individual pit-marked fish as well as growth per tank.

$$\begin{array}{c} \text{Specific growth rate }\left(\text{SGR}\right)\text{ tank or individual}\\ \;=\;\left(\frac{\text{ln}\left(\text{Final body weight }\left(\text{g}\right)\right)-\text{ln}\left(\text{Initial body weight}\left(\text{g}\right)\right)}{30\text{ days of feeding experiment}}\right)*100\\ \text{Daily feed intake}*{\text{fish}}^{-1}\left(\text{FI}\right)=\frac{\text{Recorded feed intake }*{\text{ tank}}^{-1}\;*{\text{ day}}^{-1}\left(\text{g}\right)\;}{\text{Number of fish }*{\text{ tank}}^{-1}}\\ \text{Feed conversion rate }\left(\text{FC}\right)=\left(\frac{\text{Total feed intake }*{\text{ fish}}^{-1}\left(\text{g}\right)}{\text{Body weight gain }\left(\text{g}\right)}\right) \end{array}$$

### Experimental Diets

The experimental diets were produced by dissolving CPM (Sigma-Aldrich AS, Norway) in feed oil (mixture of soybean oil and fish oil, 1:6). The feed oil was then vacuum-coated on top of a commercially available pellet (Amber Neptun for marine fish, 3 mm, Skretting, Stavanger, Norway) at a level of 1% oil inclusion. The control diet was produced correspondingly using 1% feed oil without CPM. All four batches of feed were analyzed for CPM content immediately after production and thereafter stored at −20°C until use.

### Quantification of Chlorpyrifos-methyl and 3,5,6–Trichloro–2–pyridinol

The concentration of CPM in feeds was quantified with gas chromatography using flame photometric detection by Eurofins (Eurofins Scientific, Hamburg Germany), based on the multi-residue method DFG S19 (CEN EN12393). The content of CPM in the control feed was below the limit of quantification, which was 0.01 mg/kg.

Liver and bile samples from nine fish from each treatment were pooled to three samples (pools of three fish per replicate tank). Quantification of CPM and its main metabolite 3,5,6-trichloro-2-pyridinol in liver and bile was done with ultra-high performance liquid chromatography/electrospray ionization (ESI)–tandem mass spectrometry (MS/MS) analysis. Liver samples of 0.5 g of pooled tissue were accurately weighed into a 15-ml Falcon tube. Each pool consisted of equal amounts of liver from three fish. Then, 1-ml acetonitrile (ACN):acetone (80:20) with 1% formic acid was added, and the tube was vigorously shaken by vortex for 1 min. After that, 0.5 g of MgSO_4_ was added, and the tube was immediately shaken for 1 min more. The sample was centrifuged at 6,000 rcf·g for 5 min, and, finally, 100 µl of the extract was diluted with 800-µl water and 100 µl of 25 ng/ml stable isotopically labeled internal standard (SIL-IS) solution. Finally, the diluted extract was filtered through 0.45-µm nylon filters, and 2 µl was injected into the liquid chromatography (LC)–MS/MS system. For bile samples, 400 µl (of pooled samples) of ACN:acetone (80:20) with 1% formic acid was added to 100 µl of bile in a 2-ml Eppendorf tube. The tube was vortexed for 1 min and centrifuged at 12,600 rcf·g for 5 min. Then, 250 µl of the extract was fourfold diluted with 650-µl water and 100 µl of 25 ng/ml SIL-IS solution. Finally, the diluted extract was filtered through 0.45-µm nylon filters, and 2 µl was injected into the LC-MS/MS system together with the internal-labeled internal standard mix (composed by CPM-D_6_ and TCP-^13^C_3_). The LC-MS/MS system was equipped with a triple quadrupole analyzer (TQS, Waters). Quantification was performed by means of calibration standards in solvent using relative responses to the selected SIL-IS, except for bile that was quantified using matrix-matched calibration standards. Sample identity was confirmed according to the SANTE/11813/2017, 2017 guideline (European Commission, 2017).

### RNA Isolation

Liver specimen were thoroughly homogenized on dry ice before RNA extraction using the RNeasy Plus mini kit (Qiagen, Oslo, Norway). RNA quantity and quality were determined by using the NanoDrop ND-1000 UV-Vis Spectrophotometer (NanoDrop Technologies, Wilmington, DE, USA) and the Agilent 2100 Bioanalyzer (Agilent Technologies, Palo Alto-CA, USA). The RNA integrity number (RIN value) was 9.3 ± 0.1 (mean ± SEM, *n* = 36).

### Transcriptomic Analysis

RNA-seq was used to screen for transcripts potentially affected by 30 days of dietary CPM exposure in 36 cod liver samples (*n* = 9 per treatment). For a detailed description of the RNA-seq method, see Olsvik et al. (2019). Briefly, 3-μg total RNA per sample was used as input material for the RNA sample preparations. Sequencing libraries were generated using NEBNext^®^ UltraTM RNA Library Prep Kit for Illumina^®^ (NEB, USA) following the manufacturer’s recommendations. Messenger RNA was purified from total RNA using poly-T oligo-attached magnetic beads. Fragmentation was carried out using divalent cations under elevated temperature in NEBNext First-Strand Synthesis Reaction Buffer (5X). First-strand complementary DNA (cDNA) was synthesized using random hexamer primer and M-MuLV reverse transcriptase (RNase H-). Second-strand cDNA synthesis was subsequently performed using DNA Polymerase I and RNase H. After adenylation, NEBNext Adaptor were ligated to prepare for hybridization. Library fragments were purified with AMPure XP system (Beckman Coulter, Beverly, USA). USER Enzyme (NEB, USA) was used with size-selected, adaptor-ligated cDNA before polymerase chain reaction. At last, polymerase chain reaction products were purified (AMPure XP system), and library quality was assessed on the Agilent Bioanalyzer 2100 system. The clustering of the index-coded samples was performed on a cBot Cluster Generation System using HiSeq PE Cluster Kit cBot-HS (Illumina) according to the manufacturer’s instructions. After cluster generation, the library preparations were sequenced on an Illumina NovaSeq platform, and paired-end reads were generated. Clean reads (49,654,219 ± 5,149,769, *n* = 36, mean ± st.dev.) were obtained by removing reads containing adapter, reads containing poly-N, and low-quality reads from raw data. Alignment and mapping were conducted by using TopHat2 and the Atlantic cod reference genome (deposited at ENSEMBL, about 70% of clean reads mapped to the genome). RSEM software was used to estimate gene expression levels for each sample (Li and Dewey, 2011). Clean data were mapped back onto the assembled transcriptome, and a read-count for each gene was acquired from the mapping results.

### Metabolomic Analysis

Global biochemical profiles were determined for 36 liver samples (*n* = 9 per treatment). Extracted samples were divided into five fractions: two for analysis by two separate reversed-phase/ultra-performance liquid chromatography (UPLC)–MS/MS methods with positive ion mode ESI, one for analysis by reversed-phase/UPLC-MS/MS with negative ion mode ESI, one for analysis by hydrophilic interaction chromatography/UPLC-MS/MS with negative ion mode ESI, and one sample was kept for backup. Waters ACQUITY UPLC and Thermo Scientific Q-Exactive high-resolution/accurate mass spectrometer interfaced with a heated electrospray ionization (HESI-II) source and Orbitrap mass analyzer operated at 35,000 mass resolution were utilized for all methods. The sample extract was dried then reconstituted in solvents compatible with each of the four methods. Each reconstitution solvent contained a series of standards at fixed concentrations to ensure injection and chromatographic consistency. Two aliquots were analyzed using acidic positive ion conditions. One was optimized for more hydrophilic compounds, while the other one was optimized for more hydrophobic compounds. The third aliquot was analyzed using basic negative ion optimized conditions using a separate dedicated C18 column, while the fourth aliquot was analyzed via negative ionization. Peaks were quantified using area under the curve. The final data were normalized in terms of raw area counts. Instrument variability was 3% for internal standards, and total process variability for endogenous metabolites was 9%. Identification of known chemical entities was based on comparison with metabolomic library entries of purified standards.

### Plasma Parameters

Cortisol levels, cholinesterase activity, ALAT, ASAT, and total protein levels were determined in 36 plasma samples (*n* = 9 per treatment). Plasma cortisol was determined with solid-phase enzyme-linked immunosorbent assay using the Demeditec DEH3388 kit and the manufacturer’s protocol (Demeditec Diagnostics GmbH, Kiel-Wellsee, Germany) and a microplate reader at 450 nm (Sunrise absorbance reader, TECAN, Salzburg, Austria), using a standard curve of 10–800 ng/ml. Plasma cholinesterase activity (mainly butyrylcholinesterase), ALAT, ASAT, and total protein were determined using the Maxmat Biomedical Analyser (SM1167, Maxmat S.A., France) with Maxmat reagents and the appropriate calibrators, standards, and controls. Plasma cholinesterase activity was determined with a colorimetric assay. ALAT and ASAT were quantified with a kinetic UV method (an International Federation of Clinical Chemistry and Laboratory Medicine method without pyridoxal phosphate). Total protein was quantified with a biuret method. All determinations were carried out in duplicates.

### Liver 7-Ethoxyresorufin-O-Deethylase Activity

Thirty-six liver samples were analyzed for EROD activity (*n* = 9 per treatment). The preparation of microsomal fractions for EROD activity assay was conducted as described by Nilsen et al. (1998), with minor modifications as outlined by Sanden et al. (2018). In brief, 250 to 300 mg of liver tissue was used to prepare the microsomal fractions. The microsomal fraction per well (10 μl) was added and incubated in 0.98-ml EROD buffer with 5-μl 7-ethoxyresorufin solution and 5-μl nicotinamide adenine dinucleotide phosphate (NADPH) solution for 12 min. Two wells with 0.99-ml EROD buffer, 5-μl 7-ethoxyresorufin solution, and 5-μl NADPH solution were prepared for background subtraction. Production of resorufin was quantified as fluorescence change over time (Victor TMX5 2030 Multilabel Plate Reader, Perkin Elmer, Upplands Väsby, Sweden). Enzyme activity (micromole per minute per milligram protein) was calculated as described by Ganassin et al. (2000). For calculation of microsomal fraction total protein concentration, a Pierce 660-nm protein assay was used according to the manufacturer’s protocol (Life Technologies, Oslo, Norway).

### Hypoxia Stress Test

Eighteen fish (*n* = 6 per replicate tank) per treatment were randomly selected and netted into a tank with the same dimensions as the experimental tanks (total 72 fish). After a 4-h period of undisturbed condition, the water inlet was gently closed to generate gradually reduced O_2_ concentration in the water. O_2_ saturation was constantly monitored and recorded every minute during the trial. At loss of equilibrium, fish were removed and time, PIT-ID, and O_2_ saturation recorded, defining the critical low oxygen concentration for the individual fish (LOE_hyp_). Removed fish were transferred to tanks with fully O_2_ saturated water and regained equilibrium and normal swimming behavior within minutes.

### Statistics

One-way analysis of variance (ANOVA) with Holm–Šidák’s post hoc test was used to test whether the treatment effects were significant. Brown–Forsythe and Bartlett’s tests were used for evaluation of homogeneity of variances, and the values were log-transformed before ANOVA if deemed necessary. Nonparametric Spearman’s rank correlation was used to search for relationship between two ranked variables. A parametric survival test (generalized linear model), using time to LOE_hyp_ (LogNormal) and fish weight as input variables, was used to test for differences among treatments in response to gradual hypoxia.

Differential expression analysis of two treatment groups for the RNA-seq data was performed on gene level raw read count matrix using the Bioconductor DESeq2 package (Love et al., 2014) implemented in the program “R” (http://cran.r-project.org/). Genes with an adjusted p-value (Benjamini and Hochberg, 1995) as determined by DESeq2 (p-adj < 0.05) were considered differentially expressed. Genes differentially expressed with a more relaxed cutoff (p-adj < 0.1) were used for comparison with metabolites in pathway enrichment analysis in Ingenuity Pathway Analysis (IPA) (Qiagen, Redwood City, CA, USA) and MetaCore (Thompson Reuters, Genego, St. Joseph, MI, USA). Hierarchical cluster analysis, using gene-level normalized count matrix (after regularized log transformation) and metabolites, was performed using Qlucore Omics Explorer 3.3 (Qlucore, Lund, Sweden). Gene set enrichment analysis (GSEA) was performed using human orthologs to Atlantic cod Ensembl genes as identifiers, using the gene sets Hallmark, Kyoto Encyclopedia of Genes and Genomes (KEGG), and Reactome and Gene Ontology Biological Process (GO BP) in GSEA Molecular Signatures Database (msigdb.v6.2) (Subramanian et al., 2005). The genes were ranked based on adjusted p-values from DESeq2 analysis, and the pre-ranked option of GSEA was performed.

ANOVA contrasts and Welch’s two-sample t-tests were used to identify metabolites that differed significantly between experimental groups (*p* < 0.05). Values were log-transformed before statistical analyses. Missing values were replaced with substituted values using imputation. Statistical analyses of the log-transformed data were done with “R.” Functional pathway analyses were generated using IPA and MetaCore.

Benchmark dose (BMD) analysis was performed on the responses of the dietary exposures according to the EFSA’s guidance document (EFSA, 2017b). Data were fitted on two model families (exponential and Hill), by using the EFSA web-based statistical model module (Proast, version 66.38 https://shiny-efsa.openanalytics.eu/app/bmd). Akaike information criterion (AIC) was used to assess significant fitted models. The difference of 2 in AIC values was used to accept a model as significant (EFSA, 2017b). BMD models were significant when the AIC of the model was lower than the AIC of the null model (no dose–response) -2 (AIC < AICnull - 2), and the model with lowest AIC (AICmin) was lower than the AIC of the full model +2 (AICmin < AICfull + 2) (EFSA, 2017b).

Model averaging was performed for continuous data as available in the current version of Proast. The 90% lower and upper confidence intervals for the BMD (BMDL and BMDU, respectively) were estimated including bootstrap with a standard of 200 Bootstraps. The BMDL is defined as the dose not expected to give an adverse effect. A 5% change in the benchmark response (BMR) was used as starting point for model fitting of apparent adverse effects (EFSA, 2017b) such as reduced growth, hypoxia tolerance, and cholinesterase inhibition. For markers of exposure effects on liver (transcriptomic and metabolites), the BMR was expanded, and the BMDL for BMRs of 20% changes was considered (BMDL_20_).

## Results

### Survival and Growth

Of the 276 fish used in this experiment (23 per tank), one fish from one of the control tanks died during the treatment period. No statistical difference in end weight, length, or growth was observed between the treatment groups, for the 36 fish used for downstream analyses of plasma, liver, and bile parameters (*n* = 9 per treatment), or the 72 fish used in the hypoxia stress test (*n* = 18 per treatment) (one-way ANOVA, **Table 2**). For the 36 fish used in the main experiment and the 72 fish used in the hypoxia stress test, there were significant differences between the control + low group and the medium + high group (t-test, 36 fish, *p* = 0.0049; 72 fish, *p* = 0.0027), with larger weight gain in the medium + high fish group. Growth, expressed as tank SGR, however showed a significant dose–response with a BMDL of 125 µg/kg (**Table 3**). Tank SGR also correlated significantly with exposure dose, end weight, liver and bile CPM, bile TCP, and total protein in plasma (Spearman’s rank correlation analysis, *p* < 0.05).

**Table 2:** Fish weight (g), total length (cm), and growth (g) after 30 days of dietary exposure to chlorpyrifos-methyl. Overall ANOVA showed significance for growth (weight gain) for the 36 fish group (*p* = 0.0497) and the 72 fish group (*p* = 0.0251), but the Holm–Šidák’s multiple comparisons test showed no significance between the treatment groups. Mean ± SEM.

Group	n	Control			Low			Medium			High		
		Weight	Length	Growth	Weight	Length	Growth	Weight	Length	Growth	Growth	Length	Growth
Liver, bile and plasma parameters (36 fish)	9	233 ± 13	28.8 ± 0.4	25 ± 7	207 ± 21	27.8 ± 0.7	22 ± 8	229 ± 18	27.7 ± 0.6	43 ± 5	224 ± 13	28.3 ± 0.3	44 ± 7
Hypoxia stress test (72 fish)	18	213 ± 10	27.8 ± 0.4	31 ± 5	214 ± 11	27.7 ± 0.4	27 ± 5	209 ± 9	27.5 ± 0.4	42 ± 4	210 ± 10	27.5 ± 0.4	43 ± 4

**Table 3:** Benchmark doses (BMD), lower and upper 90% confidence interval [BMDL and BMDU (mg CPM/kg), respectively], ratio BMDU to BMDL (BMDU/BMDL) for relevant responses in Atlantic cod fed graded levels of chlorpyrifos-methyl for 30 days (mg CPM/kg). For parameter abbreviations, see main text.

Whole body parameters	BMDL_05_	BMDU_05_	BMDU/BMDL
Weight	none	none	
Length	none	none	
SGR individual	none	none	
SGR tank	0.125	18	144
FCR tank	none	none	
Feed intake	none	none	
Plasma parameters	BMDL_05_	BMDU_05_	BMDU/BMDL
Cholinesterase	5.55e-05	16,3	293694
Cortisol	none	none	
ASAT	none	none	
ALAT	none	none	
Total protein	2.58	25.8	10.0
Pesticide residues	BMDL_05_	BMDU_05_	BMDU/BMDL
Liver CP	none*	none*	
Liver TCP	none*	none*	
Bile CP	1.01	3,09	3,1
Bile TCP	0.08	2.24	27,1
Metabolites	BMDL_05_	BMDU_05_	BMDU/BMDL
S-methylglutathione	0.24	2.53	11
Glutarylcarnitine	0.0863	2.78	32
Lactate	0.0481	6.36	132
Creatinine	0.061	2.39	39
DiHOME	0.0624	20.3	325
17-HDoHE	1.02	19.7	19
Gene expression	BMDL_05_	BMDU_05_	BMDU/BMDL
*gsta2*	0.6	41.8	73
*gstz*	0.547	28.9	53
*mgst1*	0.371	121	326
*gpx1*	0.553	262	474
*cyp2u1*	0.1	16.3	157
*cyp2x9*	0.3	14.2	43

No difference full and regression model AICmin < AICfull + 2. indicated with “*”.

### Chlorpyrifos-methyl and 3,5,6–Trichloro–2–pyridinol Accumulation in Liver and Bile

**Table 4** shows the accumulated levels of CPM and the major metabolite TCP in liver tissue and bile after 30 days of dietary exposure. Both the liver and bile showed a dose-dependent accumulation of CPM and TCP, with significantly elevated concentrations in the high-treatment group compared with those in the control (one-way ANOVA, *p* < 0.05). Liver tissue in fish exposed to the highest concentration contained high levels of CPM, while bile from fish in the high-dose group contained high levels of TCP.

**Table 4:** Accumulated levels of chlorpyrifos-methyl and its metabolite TCP in liver (ng/g) and bile (ng/ml) of Atlantic cod exposed to the pesticide for 30 days.

	Control	Low (0.5 mg/kg)	Medium (4.2 mg/kg)	High (23.2 mg/kg)
Liver				
Chlorpyrifos-methyl	2.2 ± 0.6	50.4 ± 12.7	382.7* ± 25.7	4007* ± 426.3^b^
TCP	0.9 ± +0.4	13.3 ± 1.8	15.5 ± 8.2	66.1 ± 9.8^b^
Bile				
Chlorpyrifos-methyl	6.2 ± 0.2	6.1 ± 0.1	10.0 ± 0.4	38.0 ± 5.4^b^
TCP	3.5 ± 1.0	5.1 ± 2.3	47.3 ± 8.7	866.7* ± 265.0^b^

*Based on partly/fully estimated concentration >> highest validation level (100 ng/g or 100 ng/ml).

Measured concentrations of chlorpyrifos-methyl in the experimental diets are given in the column captions. The values for liver and bile represent pools of three animals. Three pooled samples were analyzed per treatment (n = 3). In total, 12 liver and 12 bile samples were analyzed for chlorpyrifos-methyl and TCP. The letter b denotes significant difference from the controls (one-way ANOVA). Mean ± SEM.

### Plasma Parameters

Parameters determined in plasma are shown in **Table 5**. Cortisol was significantly elevated in plasma from Atlantic cod exposed to the highest dose (one-way ANOVA, *p* = 0.0343) compared with that of the control. Plasma cholinesterase was significantly lower in fish from all exposure groups compared with that of the control (low, *p* = 0.0457; medium, *p* = 0.035; high, *p* = 0.041). Plasma cholinesterase specific activity (enzyme activity normalized to total protein, micromole per minute per milligram) showed significance only for the highest dose (*p* = 0.0293). For ALAT, there was a significant difference between the low- and medium-dose groups but no difference between controls and exposed fish. For the other plasma parameters measured (ASAT and total protein), no significant effects were observed after 30 days of exposure.

**Table 5:** Plasma parameters measured in Atlantic cod exposed to chlorpyrifos-methyl for 30 days. Overall one-way ANOVA p-values are given in the table.

Plasma parameter	ANOVA	Control	Low	Medium	High
Cortisol (ng/ml)*	*p* = 0.0476	12 ± 5^a^	40 ± 11^a^	43 ± 9^a^	63 ± 16^b^
Cholinesterase (units/ml)	*p* = 0.0043	628 ± 18^a^	575 ± 11^b^	553 ± 11^b^	572 ± 14^b^
ALAT (units/ml)**	*p* = 0.0219	6.2 ± 1.9^ab^	4.9 ± 0.9^a^	11.4 ± 2.3^b^	5.7 ± 1.0^ab^
ASAT (units/ml)	*p* = 0.4746	106 ± 21	84 ± 8	124 ± 19	106 ± 19
Total protein (g/dl)	*p* = 0.0703	4.2 ± 0.9	4.1 ± 1.3	4.2 ± 1.1	4.4 ± 0.7

*Two outliers removed from the control group [ROUT (Q = 1%)]. One missing value from the high group.

**One outlier removed from the control group and one from the high group.

Letters a and b denote significant differences. Mean ± SEM. n = 9.

### Liver 7-Ethoxyresorufin-O-Deethylase Activity

**Figure 1:**
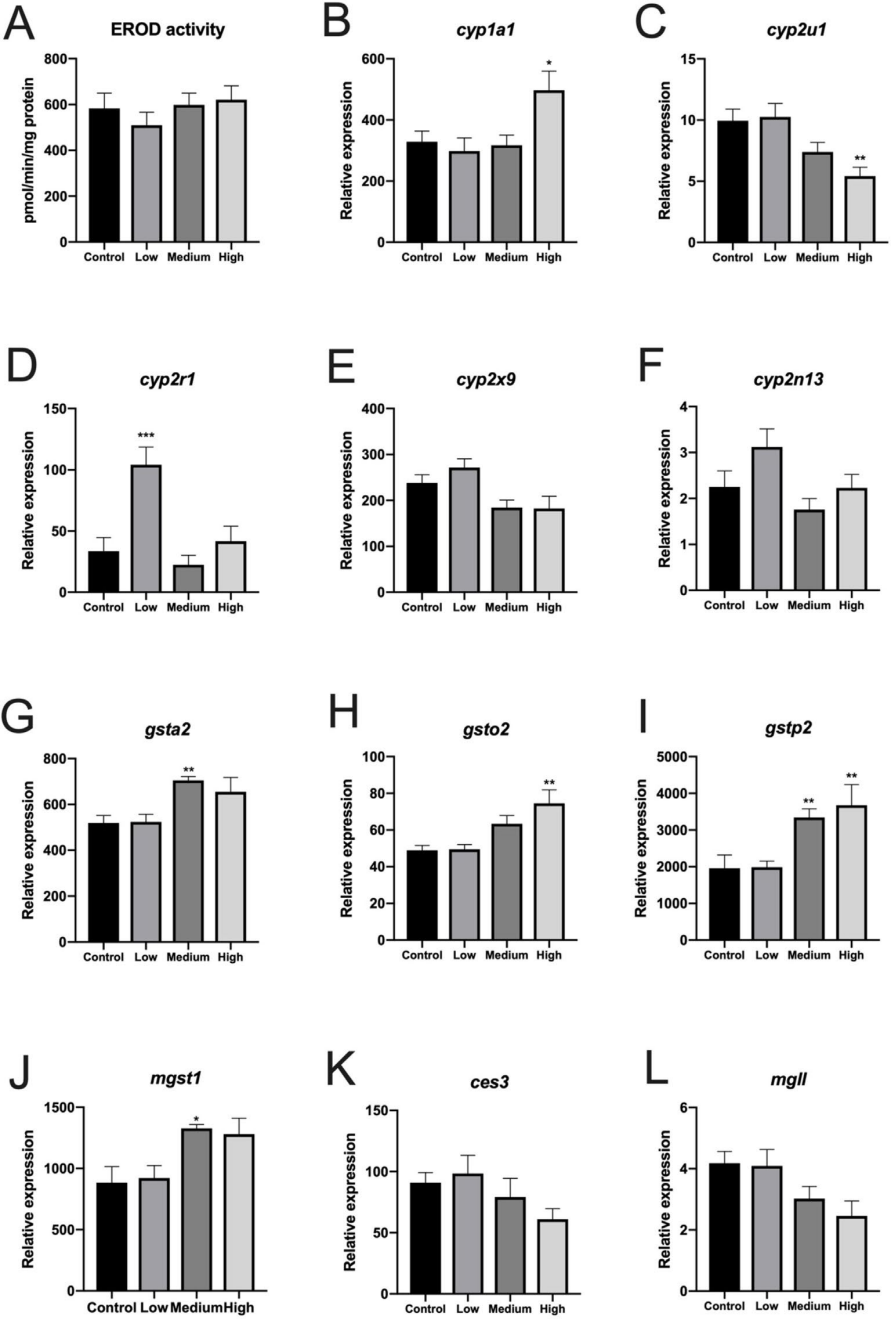
7-Ethoxyresorufin-O-deethylase activity and expression of selected genes (RNA-seq data) in liver of Atlantic cod exposed to three concentrations of chlorpyrifos-methyl for 30 days. **(A)** EROD activity, **(B)***cyp1a1*, **(C)***cyp2u1*, **(D)***cyp2r1*, **(E)***cyp2x9*, **(F)***cyp2n13*, **(G)***gsta2* (resemble human *gsta1* and zebrafish *gstal*), **(H)**, *gsto2*, **(I)***gstp2*, **(J)***mgst1*, **(K)***ces3*, and **(L)***mgll*. *n* = 6. B–L show relative transcript values based on fragments per kilobase of sequence per million mapped reads. Mean ± SEM.

Treatment with CPM for 30 days had no significant effect on liver EROD activity (**Figure 1A**). EROD activity correlated weakly but significantly with *cyp1a1* messenger RNA in liver (Spearman’s rank correlation analysis, *p* = 0.0245, *n* = 36).

### Transcriptional Responses

**Figure 2:**
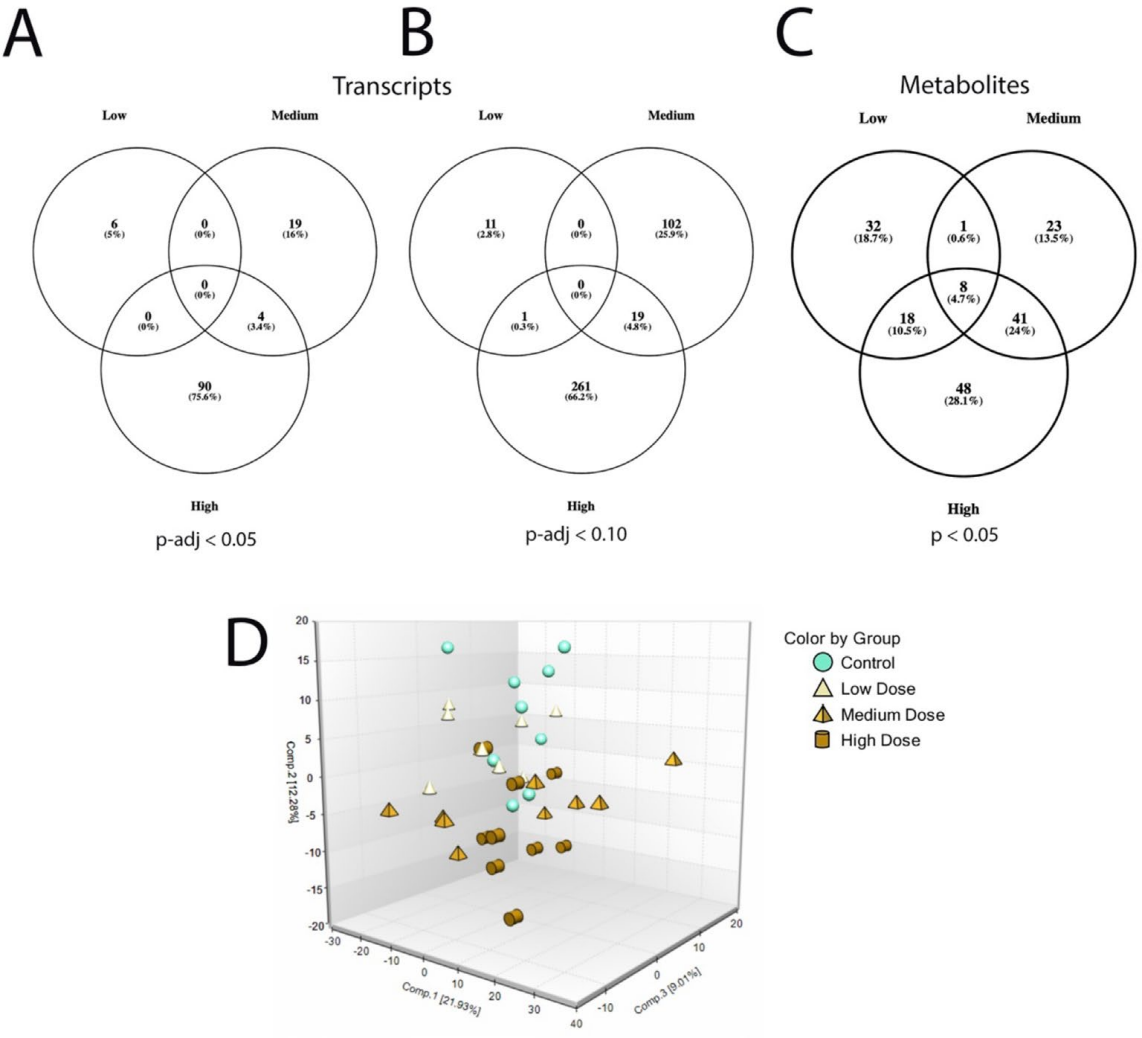
Differentially expressed transcripts and metabolites in the low-, medium-, and high-treatment groups in liver of Atlantic cod exposed to chlorpyrifos-methyl for 30 days. **(A)** Venn diagram of transcripts p-adj < 0.05, **(B)** Venn diagram of transcripts p-adj < 0.10), and **(C)** Venn diagram of metabolites p < 0.05. **(D)** Principal component analysis plot of liver metabolites.

**Figure 3:**
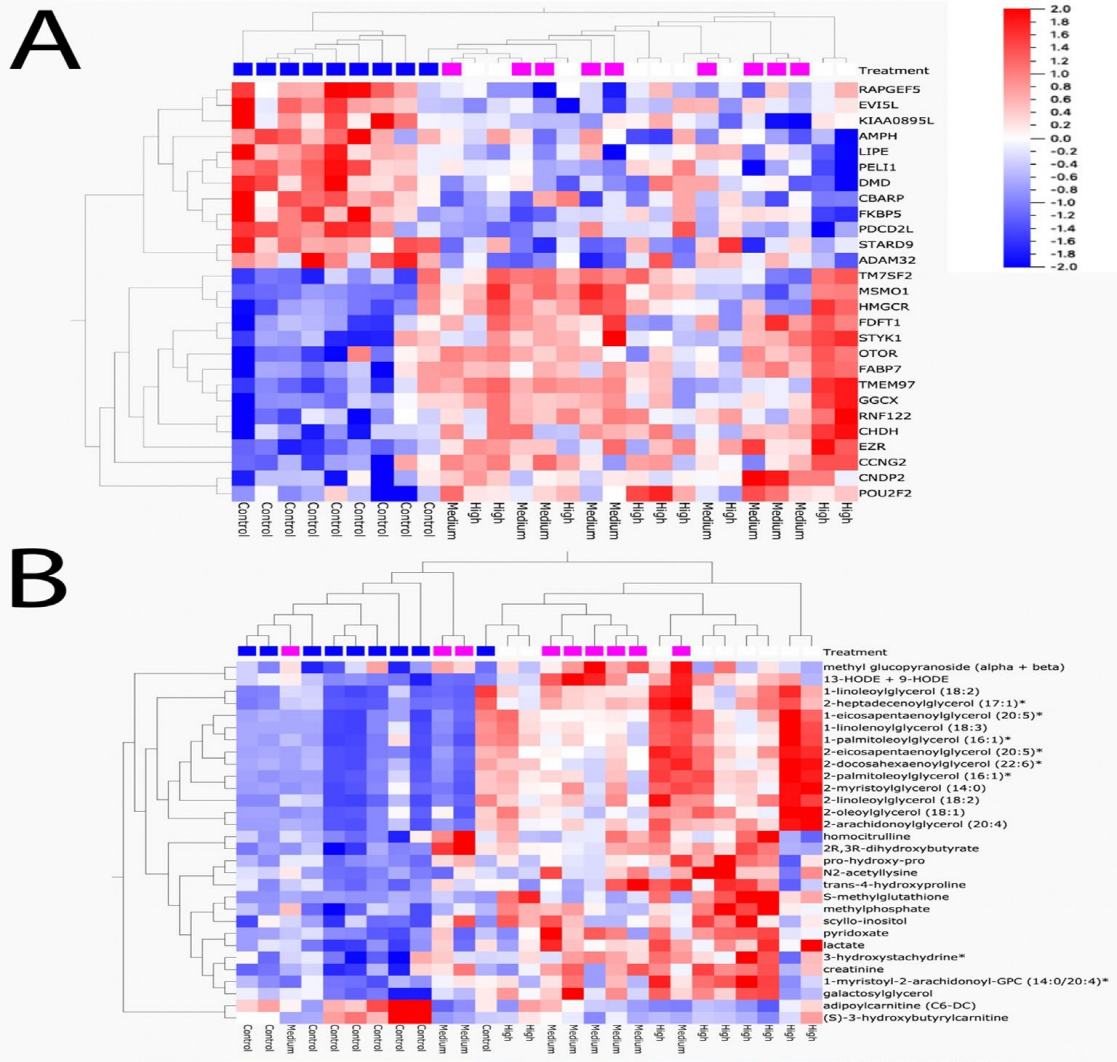
Heat map of transcripts and metabolites that best discriminate between control and chlorpyrifos-methyl-treated groups (medium + high combination). (**A**) Transcripts and (**B**) metabolites. FDR < 0.1

Sequencing output and quality are shown in **Table S1** (**Supplementary File 6**). The sequencing data have been deposited in the Gene Expression Omnibus database (accession no. GSE133594). Transcripts that were differentially regulated in liver of Atlantic cod after 30 days of being fed three levels of CPM-enriched diet are shown in **Supplementary File 1**. Most of the differentially regulated transcripts were upregulated (**Table 6**). There were few overlapping transcripts that were differentially affected by the three exposure concentrations (*p*-adj <0.05, **Figure 2A**). Transcripts with cutoff stringency *p*-adj <0.10 (**Figure 2B**) were used as input in the IPA and MetaCore analysis in combination with significant metabolites. Analyzed with one-way ANOVA, *cyp1a1* (**Figure 1B**) and four transcripts encoding glutathione-S-transferases (*gsta2*, *gsto2*, *gstp2*, and *mgst1*, **Figures 1G–J**) were significantly affected by the treatment (*p* < 0.05). Some transcripts potentially relevant for CPM detoxification are also shown in **Figure 1** (**Figures 1C–F, K**). **Figure 3A** shows a heatmap depicting expression profile of transcripts that best separate between the control and the treated (combined medium- and high-dose) groups.

**Table 6:** Number of significantly affected transcripts and metabolites in liver of Atlantic cod exposed to chlorpyrifos-methyl for 30 days using two different significance levels.

Treatment	Transcripts		Metabolites	
	*p*-adj < 0.05	*p*-adj < 0.1	*p*-adj < 0.10	*p* < 0.05
Low (0.5 mg/kg)	6 ↑ 0 ↓	10 ↑ 2 ↓	28 ↑ 10 ↓	54 ↑ 5 ↓
Medium (4.2 mg/kg)	23 ↑ 0 ↓	119 ↑ 2 ↓	36 ↑ 16 ↓	60 ↑ 13 ↓
High (23.2 mg/kg)	90 ↑ 4 ↓	264 ↑ 17 ↓	33 ↑ 18 ↓	100 ↑ 15 ↓

After filtering out genes with low (less than 10) read counts and cod genes without mapping to human orthologs (extracted from ENSEMBL database), there were 11,991 genes used as input for pre-ranked (based on *p*-adj values) GSEA. Gene sets (FDR q-value <0.25, GO, KEGG, Reactome, and Hallmark) are shown in **Supplementary File 2**. In the low-dose group, “cholesterol biosynthesis” was the most significant pathway (FDR q-value < 0.049). “Peptide chain elongation” and “ribosome” were the two most significant pathways in the medium-dose group (FDR q-value < 0.00), while “steroid biosynthesis” and “resolution of AP sites via the multiple nucleotide patch replacement pathway” (DNA repair) topped the high-dose group ranking (FDR q-value < 0.0013). There were three common gene sets, “Reactome cholesterol biosynthesis,” “KEGG steroid biosynthesis,” and “Reactome bile acid and bile salt metabolism,” enriched (FDR *q*-value < 0.25) in low-, medium- and high-treatment groups. Two elements were shared between GSEA low and GSEA high, i.e., “GO fatty acid beta-oxidation using acyl CoA dehydrogenase” and “KEGG valine leucine and isoleucine degradation.” Pathways associated with cholesterol biosynthesis were among the most significant for all dietary doses. Genes associated with cholesterol metabolism and possible effects on membrane permeability and fluidity include *acly*, *apof*, *aspa*, *cyp51a1*, *cyp7b1*, *dhcr24*, *ebp*, *fasn*, *hmgcr*, *idi1*, *lss*, *mbtps1*, *msmo1*, *scd*, *sqle*, and *tm7sf2*.

### Metabolomic Alterations

In the low, medium, and high groups, there were 59, 73, and 115 metabolites with changed levels, respectively (Welch’s two-sample t-test, *p* < 0.05) (**Table 6**). All significant metabolites are listed in **Supplementary File 3**. Contrary to the transcriptomics results, more metabolites showed overlapping responses between the three dietary doses (**Figure 2C**). Between the low group and the medium group, there were nine overlapping metabolites, while there were 48 overlapping metabolites between the medium- and high-exposure groups. Principal component analysis showed considerable overlap between the control- and low-dose groups, while greater separation was seen for the medium- and high-dose groups compared with that for the control (**Figure 2D**). Transcripts and metabolites with readable IPA identifiers linked to significantly affected diseases or functions from liver are listed in **Supplementary File 4**. **Figure 3B** shows a heatmap of metabolites that best separate between the control and the treated (medium- and high-dose) groups.

**Figure 4:**
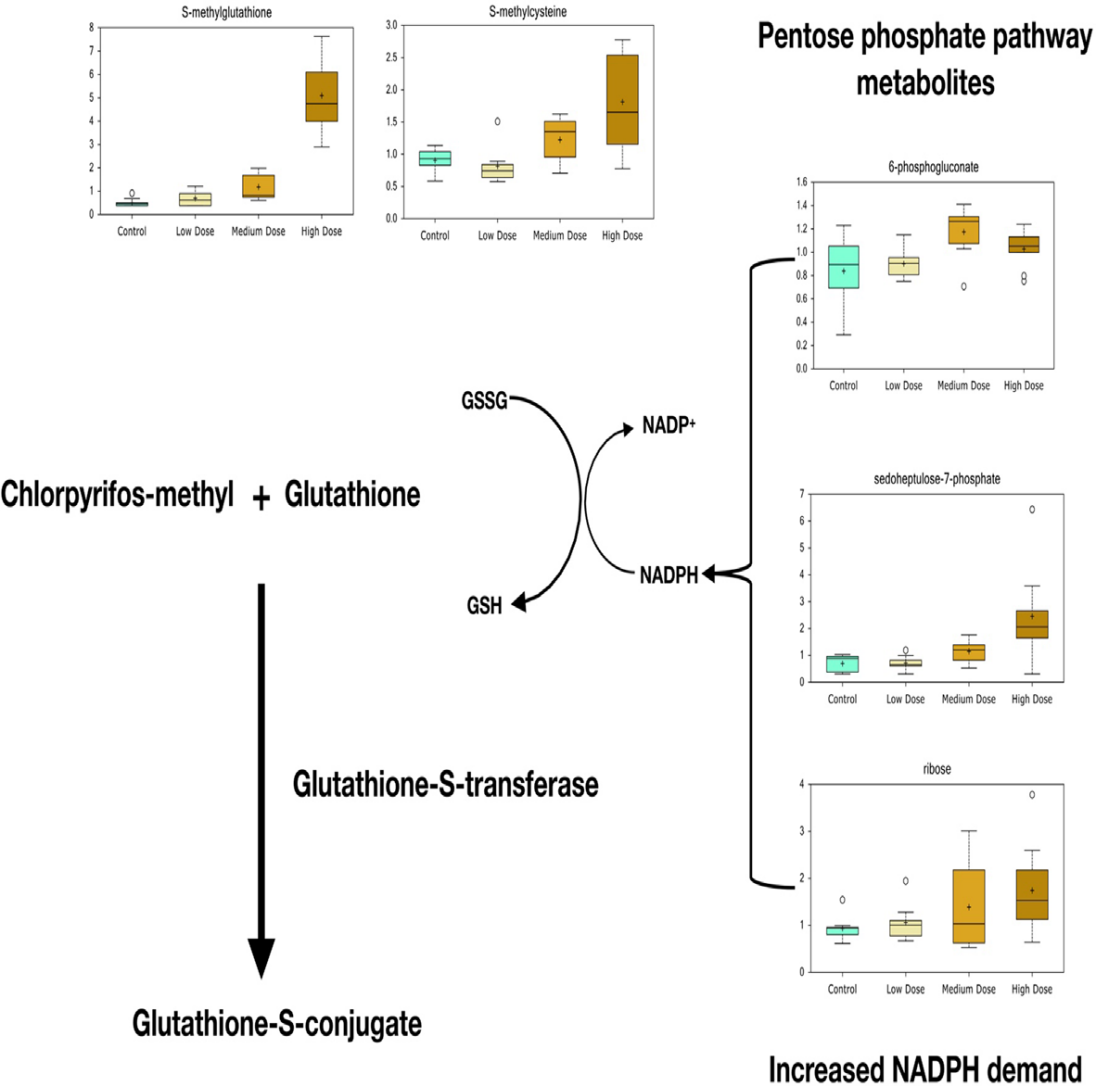
Significant metabolites predict glutathione-S-transferase-mediated detoxification of chlorpyrifos-methyl in liver of Atlantic cod after 30 days of dietary exposure. A high level of pentose phosphate pathway metabolites, including 6-phosphogluconate, sedoheptulose-7-phosphate, and ribose, predicts increased demand for NADPH for reducing power when the glutathione detoxification system is active.

According to the metabolite screening, S-methylglutathione showed 2.3- and 10-fold changes in the medium- and high-dose groups compared with those in the control, respectively (**Figure 4**). Its derivate S-methylcysteine was also increased in the treatment groups. A high level of pentose phosphate pathway metabolites, including 6-phosphogluconate, sedoheptulose-7-phosphate, and ribose, was observed at the higher doses.

**Figure 5:**
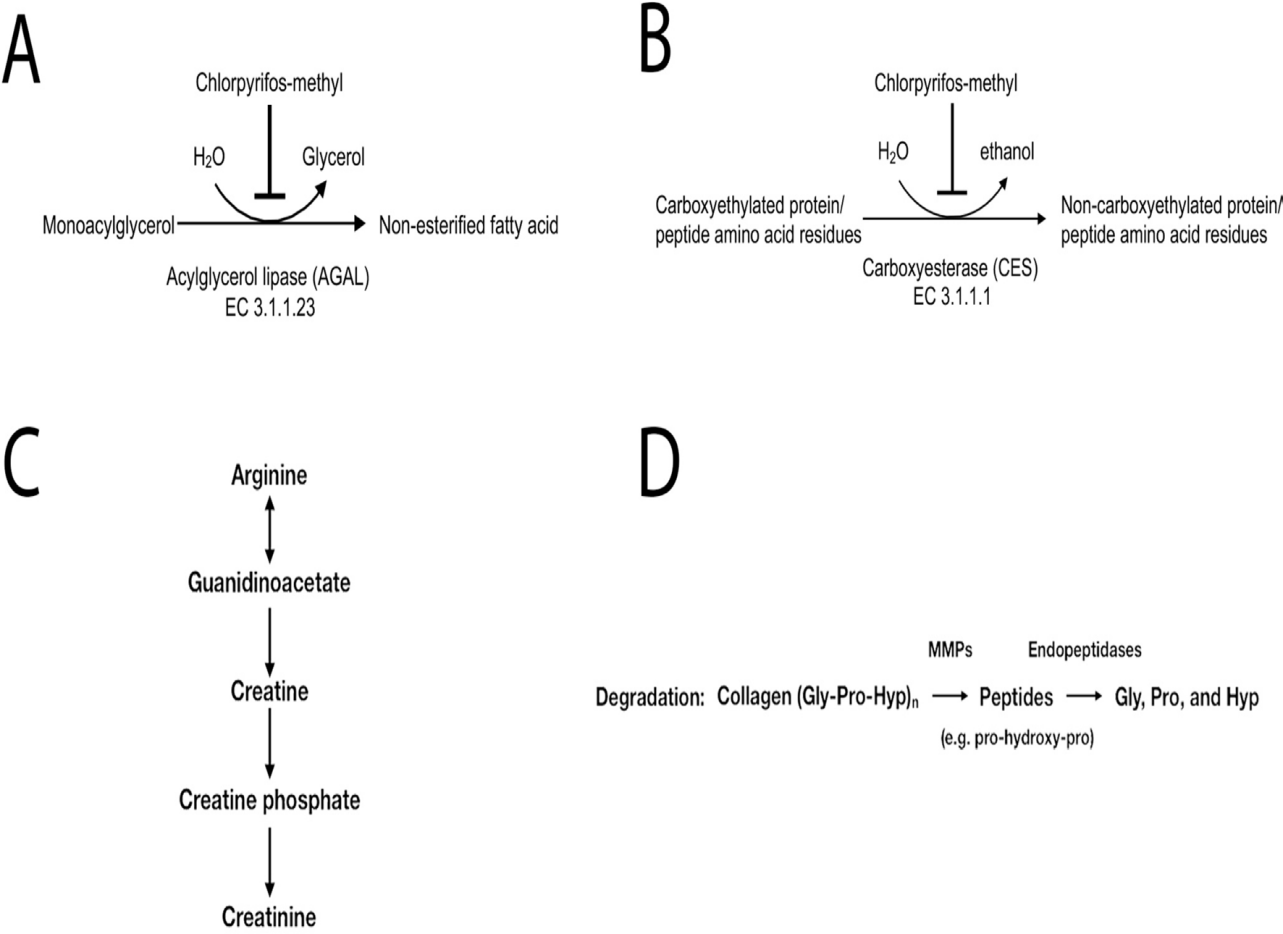
Predicted outcome of metabolites affected by chlorpyrifos-methyl exposure in Atlantic cod liver. **(A)** Inhibition of monoacylglycerol lipase (MAGL) might lead to decreased lipolysis, **(B)** inhibition of carboxylesterase (CES) might be an indicator of chlorpyrifos-methyl exposure in fish, **(C)** accumulation of creatinine and lactate indicates energy stress, and **(D)** increased collagen breakdown indicates a possible inflammation.

Inhibition of monoacylglycerol lipase (MGLL) was indicated by the elevation of nearly all monoacylglycerol (MAG) species at low and medium CPM doses and all MAG species at the highest dose. In addition, glycerol was significantly decreased at the higher dose. *Mgll*, the gene encoding MGLL, was not significantly affected at the transcriptional level by the treatment (**Figure 1L**). MGLL is responsible for the complete hydrolysis of MAGs into glycerol and free fatty acids (**Figure 5A**).

Several 1-carboxyethyl-conjugated amino acids, including 1-carboxyethylphenylalanine and 1-carboxyethylvaline, displayed dose-dependent increases with increasing CPM dose. Dose-dependent accumulation of 1-carboxyethyl-conjugated compounds such as phenylalanine, leucine, isoleucine, or valine, which are analogs to carboxyethyllysine, indicates CES inhibition (**Figure 5B**).

Levels of creatinine increased significantly in the medium- and high-CPM-dose groups compared with those in the control (**Figure 5C**). Similarly, lactate levels increased in the medium- and high-dose CPM groups compared with those in the control. Further, glutarylcarnitine, a compound derived from the mitochondrial oxidation of lysine for energy production, was significantly reduced in the medium and high-dose groups. Taken together, these changes indicate a high demand for ATP in response to the higher doses of CPM.

**Figure 6:**
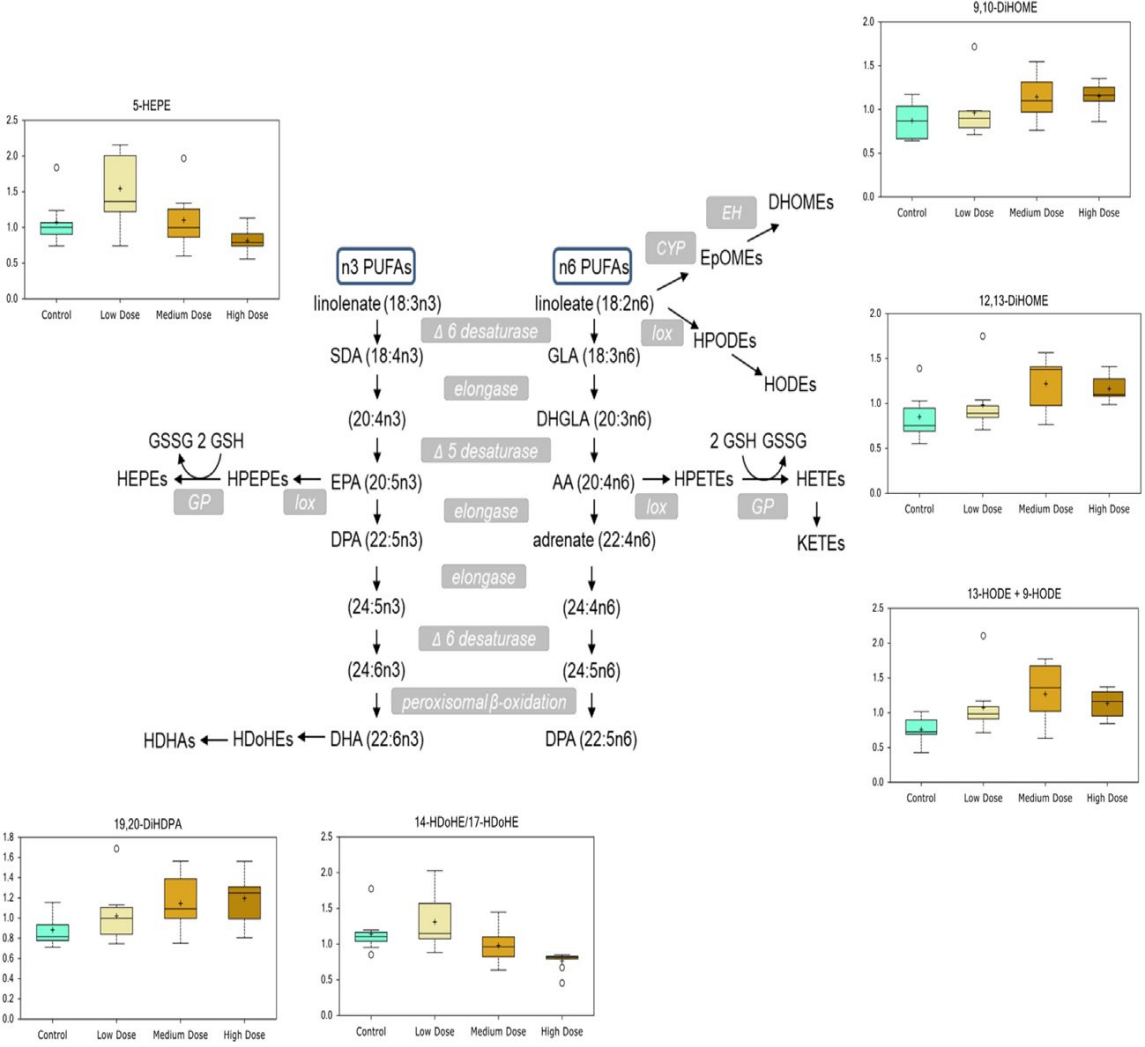
Chlorpyrifos-methyl induced changes in *n*-3- and *n*-6-polyunsaturated fatty acid-derived lipid mediators in Atlantic cod liver.

Several inflammatory markers and lipid mediators increased in response to CPM exposure. Collagen breakdown is mediated by matrix metalloproteinases (MMPs) whose expression is increased by pro-inflammatory cytokines (**Figure 5D**), and the increase of free proline-hydroxyproline (pro-hydroxy-pro), as well as trans-4-hydroxyproline in the medium- and high-dose groups, fits with possible inflammation-mediated induction of collagen breakdown. Lipid mediators derived from *n*-6 and *n*-3 polyunsaturated fatty acids (PUFAs), such as linoleate (LA), arachidonic acid (ARA), EPA, and DHA, influence inflammation with *n*-6-derived mediators tending to be more pro-inflammatory and *n*-3-derived ones being more anti-inflammatory. DiHOME and HODE compounds derived from *n*-6 linoleate increased with CPM dose, whereas HDoHEs and HEPEs derived from *n*-3 PUFAs were decreased with the highest CPM dose (**Figure 6**).

### Integrated Transcriptomics and Metabolomics Pathway Enrichment Analysis

Enriched IPA diseases or functions annotation showed lipid metabolism was affected by all doses, but the medium and high doses showed much higher number of common annotations related to lipid and amino acid metabolism (**Supplementary File 4**, **Supplementary File 6**, **Figure S1**). Pathway analysis of the differentially regulated genes (*p*-adj < 0.05) using the enrichment analysis tool Enrichr (Chen et al., 2013) showed enrichment of Wikipathways, KEGG, Reactome, and GO biological processes mainly related to cholesterol and fatty acid biosynthesis, cell cycle regulation, and the related signaling pathways. These pathways were also among the top enriched in GSEA analysis (**Supplementary File 2**). Enrichment analysis in Enrichr using the genes differentially regulated by low-, medium-, and high-dose groups also showed similar results (**Supplementary File 6**, **Table S2**). MetaCore pathway maps enriched by metabolites levels differentially altered by the individual doses are shown in **Supplementary File 6**, **Figure S2**. Although there are differences in pathways significantly enriched by the different doses, lipid metabolism-related pathways were affected by all dose groups. For example, the low dose significantly affected the “Arachidonic acid production” pathway. Regulation of lipid metabolism was significantly enriched in both the medium- and high-dose groups. An integrated transcriptomics and metabolomics pathway analysis tool, IMPaLA (Kamburov et al., 2011), was applied to search for pathways enriched by the differentially expressed genes and altered levels of metabolites (**Table S3**). Similar to the MetaCore analysis, the significantly enriched (Q-joint/FDR < 0.1) pathways by both assays are related to metabolism of lipids, fatty acid, and amino acid and derivatives. **Figure S3** (**Supplementary File 6**) shows network of proteins encoding the differentially expressed genes (human orthologs) and modulated metabolites generated using the STICTH database (Szklarczyk et al. 2016). Subnetworks of the enriched pathways such as cholesterol biosynthesis indicate the interacting proteins and the metabolites involved.

### Hypoxia Stress Test

**Figure 7:**
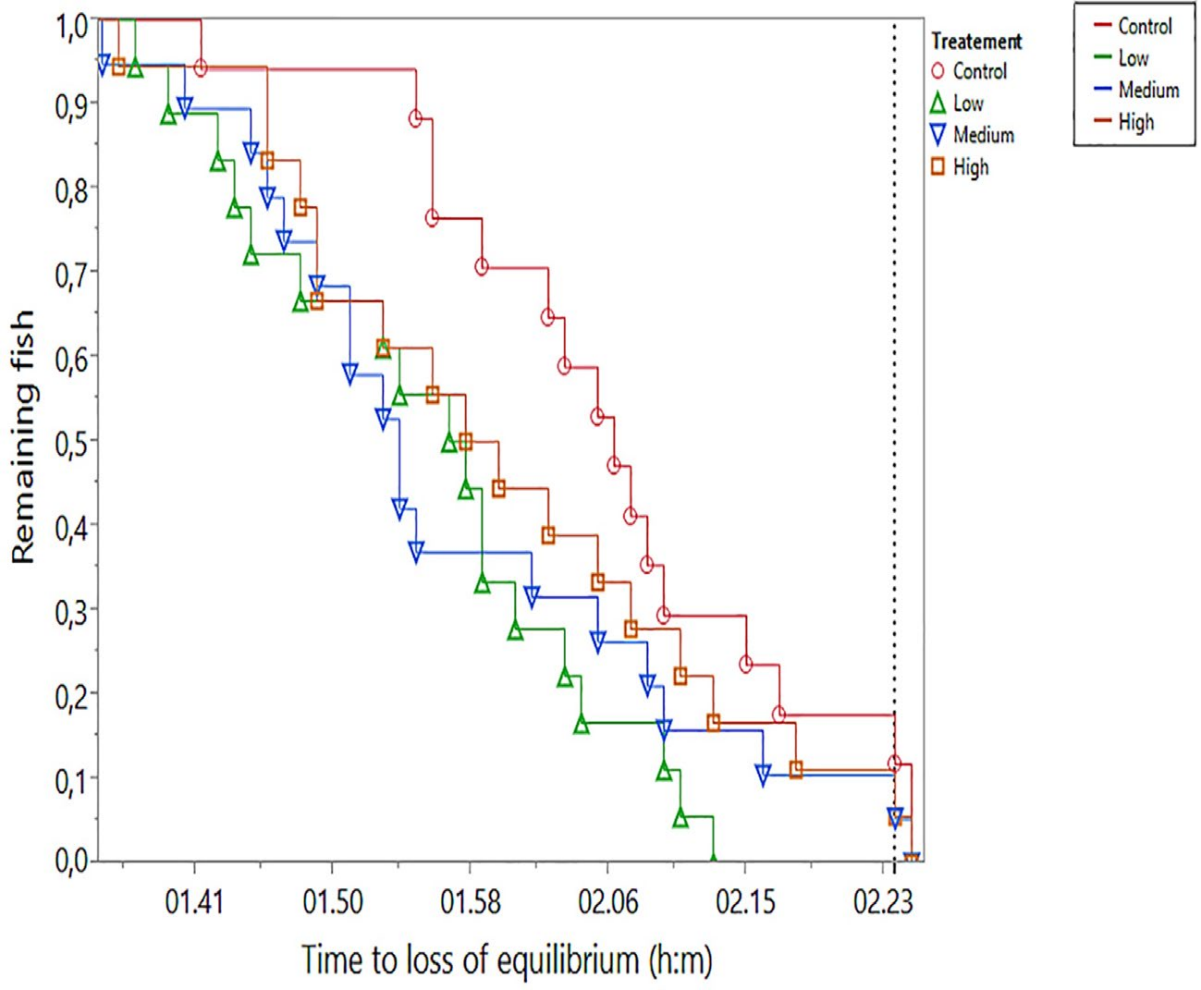
Time to hypoxia-induced loss of equilibrium (LOE_hyp_) in the four experimental treatments. Oxygen saturation during the period of LOE_hyp_ decreased gradually from 23 to 17% saturation. The vertical dotted line indicates termination of the test, at which point seven (3, 0, 2, and 2 in control, low-, medium-, and high-treatment groups, respectively) fish remained in the tank. Remaining fish were assigned time of termination and 17% O_2_ saturation.

Reduction in oxygen saturation occurred gradually, starting at 77% (16.2 kPa). Loss of equilibrium in hypoxia (LOE_hyp_) occurred over a time interval of 50 min, starting 1 h 34 min after initiation of the test (**Figure 7**). Seven fish remained at the termination of the test, which were given the time of termination as their LOE_hyp_. LOE_hyp_ occurred at relatively stable oxygen saturation levels in the range from 23 to 17% (4.8 to 3.6 kPa), and no significant difference (one-way ANOVA) between treatments in O_2_ saturation at LOE_hyp_ was found. Controls had the lowest mean LOE_hyp_ O_2_ at 18.1 ± 1.4 (mean ± SD), and low, medium, and high groups had values of 18.9 ± 1.8, 19.2 ± 1.3, and 18.8 ± 1.4, respectively. The parametric survival model showed a significant difference between groups (*p* = 0.014, whole model test) with the control group having a significantly longer time to LOE_hyp_ compared with all exposed groups. The treatment effect (*p* = 0.008) was highly significant, whereas the weight effect (*p* = 0.259) was not significant. The interaction effect (treatment * weight) was statistically significant (*p* = 0.047).

### Benchmark Dose–Response Analyses

Parameters analyzed with BMD modeling as well as BMDL and BMDU values are presented in **Table 3**. For several plasma biochemistry, liver metabolite, and liver gene expression parameters, a BMDL could be assessed, as the dose–response model was significantly better than the response model that predicts no dose–response (null model) (AIC < AICnull-2), and the best-fitted dose–response model (lowest AIC) was better than the full response model (AICmin < AICfull + 2) (**Table 3**). For final weight, length, individual SGR, tank feed intake, and feed conversion (FC) rate and LOE_hyp_, no BMDL could be established because none of the fitted dose–response models were significantly better than the null model (AIC > AICnull - 2, indicated with “none” in **Table 3**). Only for tank SGR, a significant BMDL could be established. In addition, for liver CPM and TCP levels, the best fitted BMDL model was not significantly different from the full model (indicated with “*” in **Table 3**).

For several parameters with a significant BMDL (e.g., plasma cholinesterase, tank SGR, liver metabolite lactate, and DiHome and gene expression *mgst1*, *gpx1*, and *cyp2x9*), a large variation in the BMDL and BMDU was seen as the BMDL/BMDU ratio was higher than 100 (**Table 3**). This large variation in the upper and lower 90% confidence intervals indicates a high uncertainty in BMD model assessment for these parameters. For parameters with a lower BMDU/BMDL ratio of <30, the lowest BMDLs were for plasma protein, bile CPM and TCP, and liver metabolites methylglutathione and 17HdoHE, with a BMDL_05_ or BMDL_20_ of 2.58, 1.01, 0.08, and 0.24, 1.02 mg CPM/kg feed, respectively. The BMDLs for the selected gene expression parameters had a higher uncertainty (BMDU/BMDL ratio >40) than those for liver metabolites, but the lowest BMDLs with highest certainty (BMDU/BMDL >100) were for *gsta2*, *gstz*, and *cyp2x9* with a BMDL_20_ ranging from 0.1 to 0.5 mg CPM/kg feed.

## Discussion

This study demonstrates that Atlantic cod, a species found in high abundance adjacent to Atlantic salmon fish farms, may accumulate considerable amounts of organophosphorus compounds in the liver after continuously feeding on uneaten pellets containing traces of agricultural pesticides. Compared with Atlantic salmon, Atlantic cod accumulates much higher levels of CPM in the liver after dietary exposure. In Atlantic salmon exposed to 8 mg/kg for 67 days (51 µg/kg fish per day) (Sanden et al., 2018), the level of CPM in the liver was 5.1 ± 1.6 ng/g. In this study, the accumulated level of CPM in liver of cod exposed to 4.2 mg/kg (medium dose, 5.5 µg/kg fish per day) for 30 days was 382.7 ± 25.7 ng/g or about 75 times higher. Even the lowest exposure concentration applied in this study, 0.5 mg/kg (0.68 µg/kg fish per day), induced higher bioaccumulation than 8 mg/kg for 67 days did in salmon (50.4 ± 12.7 ng/g, or about 10 times higher). This discrepancy probably in part reflects the high fat content of cod liver compared with that of the salmon liver. While cod liver contains about 55% total fat (Morais et al., 2001), Atlantic salmon liver only contains about 6% total fat (Ruyter et al., 2006). CPM is strongly lipophilic [has an octanol/water partition coefficient (log Kow = 4.31, PubChem database)] and accumulates mainly in fat.

OPs are primarily metabolized and detoxified by phase I oxidation and hydrolysis reactions and by phase II conjugation reactions (Hodgson and Rose, 2008). CPF is metabolized in liver microsomes by CYP enzymes and esterases either to CPF-oxon or to 3,5,6-trichloro-2-pyridinol (TCP), the main metabolite (Eaton et al., 2008). The CPF-oxon released into the blood acts as a strong inhibitor of cholinesterase enzymes (Garabrant et al., 2009). Biotransformation after phase I oxidation and release of CPF-oxon into the blood was indicated in this study, as all three exposure dosages significantly inhibited plasma cholinesterase activity, and the parent compound has much lower ability to inhibit cholinesterases (Sultatos, 1994; Salazar-Arredondo et al., 2008). The levels of TCP in liver tissue and bile, which showed dose–response increases, also confirmed a substantial degree of metabolism. Compared with those of Atlantic salmon (Sanden et al., 2018), the levels of TCP in liver and bile of cod were in a similar range for CPM exposure concentrations up to 5–8 mg/kg. However, at the highest exposure concentration, bile of cod had a very high TCP level as well as a high “parent compound–metabolite ratio,” indicating an efficient metabolism, but also reflecting the much higher level of CPM accumulation in liver in cod compared with that in salmon. Rapid accumulation of CPF after prolonged dietary exposure has been shown earlier in fish and also relatively fast biodegradation and elimination during depuration (Varo et al., 2002). Because of the intermediate persistent and bioaccumulative properties, CPF does not meet the criteria to be classified as a persistent organic pollutant according to the Stockholm convention (Mackay et al., 2014).

Interestingly, EROD activity was not increased in cod liver, as opposed to the dose-dependent increase in EROD activity we observed in Atlantic salmon fed three concentrations of CPM (Sanden et al., 2018). However, *cyp1a1* was increased in liver of cod exposed to the highest dosage, and EROD activity and *cyp1a1* levels correlated positively with each other. Using *in vitro* exposure, we have previously observed induction of *cyp1a1* in Atlantic salmon hepatocytes exposed to intermediate concentrations of CPF (10 µM) but not at higher doses (Søfteland et al., 2014; Olsvik et al., 2015). Such non-monotonic *cyp1a1* dose–response curves have been reported earlier in zebrafish exposed to CPF (Jeon et al., 2016a). Prolonged dietary exposure, however, did not affect the transcription of *cyp1a1* in liver of Atlantic salmon exposed to CPM (Olsvik et al., 2019). Prolonged waterborne exposure (40 days) to CPF results in *cyp1a* induction and increased EROD activity in carp (*Cyprinus carpio*) (Xing et al., 2014). The reasons for these discrepancies are not known.

One of the most profound responses to CPM exposure according to the significantly affected metabolites was the increased levels of S-methylglutathione in liver of cod medium- and high-dose groups compared with those in the control. S-methylglutathione can be formed in demethylation reactions and is a biomarker of glutathione-S-transferase-mediated detoxification of OPs that has also been linked to CPF detoxification (Tracy and Oleary, 1991; Fujioka and Casida, 2007; Espinoza et al., 2013). In Atlantic salmon liver cells exposed to PP-methyl, another widely used OP detected in salmon feed, we have seen an even stronger elevation of S-methylglutathione after 48 h *in vitro* treatment, associated with glutathione depletion (Olsvik et al., 2017). In this study, however, no effects were seen on the levels of reduced or oxidized glutathione. Four transcripts encoding GST proteins (*gsta2*, *gsto2*, *gstp2*, and *mgst1*) found in the medium and high groups showed significant differences compared with the control, supporting GST-mediated conjugation of CPM, CPF-oxon, and/or TCP in fish. Elevated levels of S-methylglutathione was accompanied by high levels of pentose phosphate pathway metabolites. In addition to being an important carbon source of anabolic precursors for nucleic acid synthesis, the oxidative branch of the pentose phosphate pathway is a major source of reducing NADPH that is demanded to recycle and regenerate glutathione during detoxification (Stincone et al., 2015). Pentose phosphate pathway activation might therefore be associated with particularly high demand for NADPH when the glutathione detoxification system is active.

In addition to inhibition of plasma cholinesterase activity, metabolite profiling also indicates inhibition of liver CES activity. Inhibition of CES activity is a well-known effect of OPs such as CPF in fish (Wheelock et al., 2005; Leticia and Gerardo, 2008; Jeon et al., 2016b). Several 1-carboxyethyl-conjugated amino acids, including 1-carboxyethylphenylalanine and 1-carboxyethylvaline, displayed dose-dependent increases with increasing CPM dose. While no information is available about these modified amino acids [1-carboxyethylated (CE) phenylalanine, leucine, isoleucine, or valine] in the scientific literature, they bear structural similarity to the modified amino acid residues carboxyethyllysine and carboxyethylarginine that have been reported as advanced glycation end products in proteins and peptides (Soboleva et al., 2017). Although speculative, these rare carboxyethyl-conjugated compounds might be biomarkers of reduced CES activity in response to exposure to OPs.

Disruption of lipid metabolism is one of the main effects of CPF/CPM exposure according to our earlier studies with Atlantic salmon. Short-term *in vitro* exposure to these widely used OPs typically leads reduced levels of many PUFAs in salmon hepatocytes and to accumulation of MAGs (Søfteland et al., 2014; Olsvik et al., 2015). In this experiment, however, many long-chain PUFAs (e.g., eicosapentaenoic acid, LA, and docosapentaenoic acid), and also long-chain saturated (SAFAs) and monosaturated fatty acids (MUFAs), were significantly higher in liver of cod exposed to the low dose (**Table 7**) but, surprisingly, not at higher concentrations. Many fatty acids, in particular PUFAs, act as ligands for peroxisome proliferator-activated receptors (Georgiadi and Kersten, 2012). In line with this, the pronounced accumulation of nearly all MAG species at all three CPM doses predicts decreased lipolysis in cod liver. A similar accumulation of MAGs has been seen in brain and liver of mice exposed to CPF as a result of inhibition of MGLL (Medina-Cleghorn et al., 2014). Inhibition of serine hydrolases such as MGLL has been shown to be one of the main effects of OPs (Casida and Quistad, 2005; Medina-Cleghorn et al., 2014). Based on increased levels of 2-arachidonoylglycerol, 2-linoleoylglycerol and 2-oleoylglycerol, IPA core analysis predicted MGLL as the top upstream regulator in the low dose and among the predicted significant upstream regulators in the medium and high groups (**Supplementary File 5**). Alternatively, CPF may act by changing lipid homeostasis to promote lipogenesis and lipid accumulation (Darbre, 2017), as suggested by increased lipogenic transcripts *acly*, *fasn*, and *scd*.

**Table 7:** List of fatty acids that accumulated in liver of Atlantic cod low-dose group after 30 days of exposure to chlorpyrifos-methyl.

Sub-pathway	Fatty acid	Low dose increase (fold change)
Long Chain Saturated Fatty Acid (SAFA)	myristate (14:0)	1.26
	pentadecanoate (15:0)	1.32
	palmitate (16:0)	1.22
	margarate (17:0)	1.40
Long Chain Monounsaturated Fatty Acid (MUFA)	myristoleate (14:1n5)	1.35
	palmitoleate (16:1n7)	1.48
	10-heptadecenoate (17:1n7)	1.54
	oleate/vaccenate (18:1)	1.31
	10-nonadecenoate (19:1n9)	1.48
	eicosenoate (20:1)	1.43
Long Chain Polyunsaturated Fatty Acid (*n*-3 and *n*-6) (PUFA)	hexadecatrienoate (16:3n3)	1.44
	heptadecatrienoate (17:3)	1.41
	stearidonate (18:4n3)	1.47
	omega-3 arachidonate (20:4n3)	1.72
	eicosapentaenoate (EPA; 20:5n3)	1.29
	heneicosapentaenoate (21:5n3)	1.65
	docosapentaenoate (n3 DPA; 22:5n3)	1.74
	nisinate (24:6n3)	1.45
	hexadecadienoate (16:2n6)	1.47
	linoleate (18:2n6)	1.43
	linolenate [alpha or gamma; (18:3n3 or 6)]	1.30
	dihomo-linoleate (20:2n6)	1.59
	dihomo-linolenate (20:3n3 or n6)	1.55
Fatty Acid, Branched	(12 or 13)-methylmyristate (a15:0 or i15:0)	1.38
	(14 or 15)-methylpalmitate (a17:0 or i17:0)	1.44

After 30 days of dietary exposure to CPM in Atlantic salmon liver, phosphatidylinositol, palmitate, and ARA content significantly decreased with increasing CPM dose (Sanden et al., 2018). A similar response was observed in cod liver for one phosphatidylinositol, with significantly reduced levels of 1-stearoyl-2-arachidonoyl-GPI (18:0/20:4) but not for palmitate and ARA. However, a possible link to altered ARA metabolism was the observed accumulation of lipid mediators derived from LA, such as 9-HODE, 13-HODE, 9,10-DiHOME, and 12,13-DiHOME, in cod liver after CPM exposure. Arachidonic acid is synthesized from α-LA derived from LA and is found ubiquitously in plasma membranes, where it is bound to phospholipid (Hanna and Hafez, 2018). These pro-inflammatory compounds derived from *n*-6 LA also point to a greater level of inflammation, as supported by the accumulation of collagen breakdown markers pro-hydroxy-pro and trans-4-hydroxyproline in response to CPM exposure. Many of the predicted activated upstream regulators in the high-dose group, such as SREBF1/F2, SCAP, are associated with lipid metabolic processes, further underlining the impact CPF has on lipids. Taken together, this study shows that CPM has a profound effect on lipid metabolism in Atlantic cod after 30 days of dietary exposure, in line with responses seen in other fish species earlier.

Accumulation of creatinine and lactate in cod liver at medium- and high-dose CPM exposure indicates elevated energy stress. Creatinine is a key indicator of usage of energy reserves from the high-energy phosphate compound creatine phosphate (Wyss and Kaddurah-Daouk, 2000). Lactate can be generated in muscles during periods of high-energy demand and converted back to glucose in the liver. Reduced levels of glutarylcarnitine, derived from mitochondrial oxidation of lysine for energy production, points in the same direction. Enrichment analysis using a list of all significant metabolites supported elevated energy stress, with “carbohydrate metabolism, TCA and tricarboxylic acid transport” and “carbohydrate metabolism, pyruvate metabolism and transport new” being the most significant pathways. Metabolite profiling has predicted a similar outcome after short-term exposure to CPF and PP-methyl in liver of Atlantic salmon, with cells exhibiting reduced levels of the glycogen metabolites (Olsvik et al., 2015; Olsvik et al., 2017). It thus appears that these OPs have a negative impact on energy metabolism in liver of exposed fish. The exact mechanism behind the CPM-induced energy stress is not known, but it is interesting to speculate that part of the increased demand for ATP is linked to activation of the P-glycoprotein pump (cellular efflux transporter ABCB1 in mammals, ABCB4 in fish) in order to transport chemical conjugates across cell membranes. The ATP-dependent efflux pump P-glycoprotein has been implicated in the stress response to CPF (Bain and LeBlanc, 1996) and helps protect cells from the effects of a variety of xenobiotics (Fischer et al., 2013; Alharbi et al., 2017). Energy stress might also be linked to perturbations of hepatic fat metabolism, as CPF in mice has been shown to block 21 different metabolic serine hydrolases after *in vivo* exposure (Medina-Cleghorn et al., 2014).

Atlantic cod is relatively tolerant to hypoxia and may inhabit low oxygen environments (Claireaux and Dutil, 1992; Scholtz and Waller, 1992; Schurmann and Steffensen, 1992; Plante et al., 1998). We have previously shown that prolonged exposure to 45% O_2_ saturation had no impact on the oxidative stress index in juvenile Atlantic cod (Olsvik et al., 2006). After dietary exposure to CPM, reduced hematocrit level was the only blood parameter that was significantly affected in Atlantic salmon (Sanden et al., 2018). Reduced hematocrit is often observed in fish after waterborne exposure to OPs, including CPF (Koprucu et al., 2006; Mostakim et al., 2015; Ismail et al., 2018). Hematocrit was not measured in the current experiment, due to high variability reported in the literature (e.g., Audet et al., 1993; Foss et al., 2006) and high probability of stress-induced splenic red blood cell release and red blood cell swelling during handling and anesthesia before sampling (Nilsson and Grove, 1974; Gallaugher et al., 1995). In the low-dose group, one of the transcripts with *p*-adj < 0.05 was annotated to erythropoietin, indicating long-term effects on erythropoiesis (Chu et al., 2008). A reduced level of red blood cells indicates an impaired capacity to deliver O_2_ to vital organs during stress. In this work, we therefore included a hypoxia challenge test at the end of the 30-day long feeding trial, to search for possible physiological impairment and phenotypic effects of the treatment. The LOE_hyp_ test was chosen in this study as recommended by Wood (2018) as an easy and reliable indicator of hypoxia tolerance. The hypoxia test showed that cod pre-exposed to all levels of CPM had significantly poorer ability to handle acute hypoxia, mainly indicated by the time until LOE_hyp_. LOE_hyp_ levels corresponded well with literature values for Atlantic cod (Plante et al., 1998; Petersen and Gamperl, 2011). However, no clear dose–response effect could be observed, as no significant BMDL could be established. Size effects on hypoxia tolerance in fish are inconsistent and debated in the literature (Nilsson and Ostlund-Nilsson, 2008). However, the best model in this experiment included accounting for size effects but only in interaction with treatment. Parameter estimates for the weight* treatment effect were all negative, indicating larger fish had lower LOE_hyp_. Excluding weight as a variable in the model still resulted in a significant treatment effect (*p* = 0.044), indicating a consistent effect of CPM at the whole-body level manifested as reduced hypoxia tolerance.

Traditionally, toxicology studies use the no observed adverse effect level (NOAEL), based on a (sub)-chronic dose–response study with graded levels of the contaminant (Teh et al., 2004), to assess safe dietary levels of feed contaminants for animal health. The use of ANOVA to assess lowest observed adverse effect levels (LOAEL) or NOAEL leaves uncertainty as to where the toxicity threshold lies between the NOAEL and LOAEL, or even lower than the NOAEL (Rigby et al., 2010). Furthermore, the assessment of safe limits by use of NOAEL strongly depends on the chosen exposure concentrations and is often expressed as the control group (Berntssen et al., 2018). In the present study, plasma cholinesterase had a LOAEL of 0.5-mg CPM per kilogram; however, the NOAEL was the control group that did not contain detectable (<0.01 mg/kg) CPM feed levels. The threshold for safe exposure would be within a range of 10–500-µg CPM per kilogram feed. Surveillance of Norwegian commercially produced Atlantic salmon feed showed the presence of CPM in 4 of 40 analyzed feeds in 2017 (detection limit 10 µg/kg), with levels of 11–26 µg/kg (Sele et al., 2018). The exposure of cod to CPM by waste feed would be between the NOAEL of 10 µg/kg and the LOAEL of 500 µg/kg. The European Food Safety Authority (EFSA) recently recommended to use benchmark dose (BMD) models to assess safe levels in feed of supplements or contaminants instead of NOAEL (EFSA, 2017b; Berntssen et al., 2018). In addition, definitions of the difference between adverse effect, biomarkers of exposure or effect, and mode of action were clarified in a guidance document (EFSA, 2017a). By using the BMDL, a common approach in dose–response assessment is used to set a safe limit for undesirables in feed or food (EFSA, 2017b). The parameters with the best dose–response model fits (lowest BMDL and BMDU variation and AIC) and most sensitive responses (lowest BMDL) were bile TCP, plasma protein, and liver metabolites. These parameters gave a safe dietary dose for CPM-exposed fish of 80–258 µg/kg. For transcriptomic responses, which had a higher variation, the safe limits could be set on 100–500 µg/kg. Unfortunately, for plasma cholinesterase, which had a lowest adverse effect level (LOEL) of 500 µg/kg feed, the BMD assessment showed a large variation (BMDL and BMDU variation of ∼300,000) causing the use of the BMDL of 0.05 µg/kg as a safe dietary CPM level to be very uncertain. For comparison, the average BMD was 230 µg/kg. Hence, based on the BMDL modeling from this 30-day feeding trial, no adverse effect is expected in wild fish feeding on leftover pellets near salmon farms. However, it is important to keep in mind that a normal production cycle for farmed Atlantic salmon in seawater lasts for 14–24 months, depending on seawater conditions. Atlantic cod accustomed to feeding on leftover pellets may therefore be more vulnerable to longer-term continuous exposure to OPs than predicted in the current models. In addition to a residence at specific farms, frequent movements of wild fish to adjacent farms have also been observed (Uglem et al., 2009). Wild fish might therefore be continuously exposed to foodborne contaminants for an extended period of time.

In conclusion, this study was conducted to gain insight into the consequences of prolonged exposure to the insecticide CPM in Atlantic cod, a fish species found in abundance near fish farms in Norway. Aquafeeds for Atlantic salmon contain trace amounts of OPs that may potentially be damaging to wild fish after chronic exposure. In addition to plasma cholinesterase inhibition, CPM exposure induced a range of responses reflecting detoxification by GSTs, inhibition of MGLL, potential inhibition of CES, and increased demand for ATP, as well as secondary inflammatory responses. Based on the assessed BMDL safe feed limit of 80–100 µg/kg feed and the highest levels observed in commercial salmon feed of 26 µg/kg in 2017, the potentially harmful effect of cod consuming feed waste is limited. However, additional dose–response studies are needed to assess safe feed exposure on sensitive parameters such as cholinesterase as well as extrapolating the current data to long-term effects on population dynamics of wild fish.

## Data Availability

The RNA-seq datasets generated for this study can be found in the Gene Expression Omnibus https://www.ncbi.nlm.nih.gov/geo/query/acc.cgi?acc=GSE133594.

## Ethics Statement

The experiment complied with the guidelines of the Norwegian Regulation on Animal Experimentation and EC Directive 86/609/EEC. The National Animal Research Authority in Norway approved the protocol (FOTS approval ID 15057).

## Author Contributions

PO conceived the project. PO, AKL, and TK designed the experiment with advice from MB and MS. PO, AKL, MB, and TK performed the experimental work. PO and FY analyzed and interpreted the transcriptomics and metabolomics data. TK interpreted the hypoxia test results, while MB did the benchmark dose modeling work. PO wrote the manuscript with support from AKL, MB, FY, and TK. AG and OAK provided funding acquisition and project administration. All authors critically revised and approved the final manuscript.

## Funding

This work was financed by the Norwegian Research Council (iCod2 project 244564 and dCod 1.0 project 248840) and the Institute of Marine Research.

## Conflict of Interest Statement

The authors declare that the research was conducted in the absence of any commercial or financial relationships that could be construed as a potential conflict of interest.

## Supplementary Material

Supplementary File 1 List of significant transcript.

Supplementary File 2 List of gene sets from GSEA analysis.

Supplementary File 3 List of significant metabolites.

Supplementary File 4 List of diseases functions and annotations from IPA analysis.

Supplementary File 5 Predicted upstream regulators.

Supplementary File 6 Pathway enrichment based on genes and metabolites.
