# Supplementary material for: Effects of Agricultural Pesticides in Aquafeeds on Wild Fish Feeding on Leftover Pellets Near Fish Farms
Source: Front Genet. 2019 Sep 26;10:794. doi: 10.3389/fgene.2019.00794 (PMC6775492; doi:10.3389/fgene.2019.00794)
Supplement: Supplementary File 6 — Pathway enrichment based on genes and metabolites. [file DataSheet_1.docx]

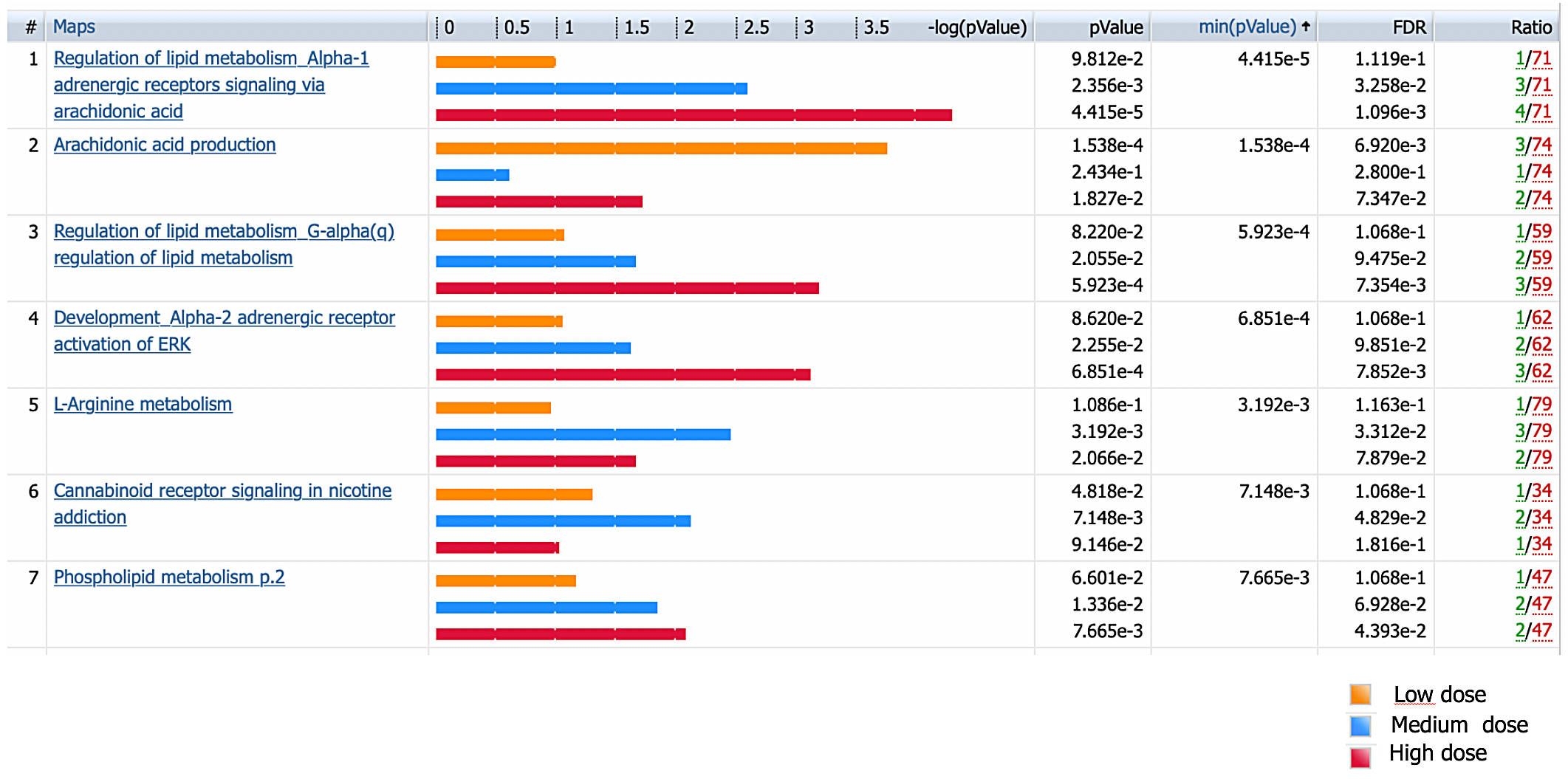


Figure S1: Enriched MetaCore Pathway Maps by metabolites levels differentially modulated (*p*<0.05) by Low, Medium and High doses of CPM. Only the top enriched maps with FDR q-value<0.05 in at least one dose group are shown.


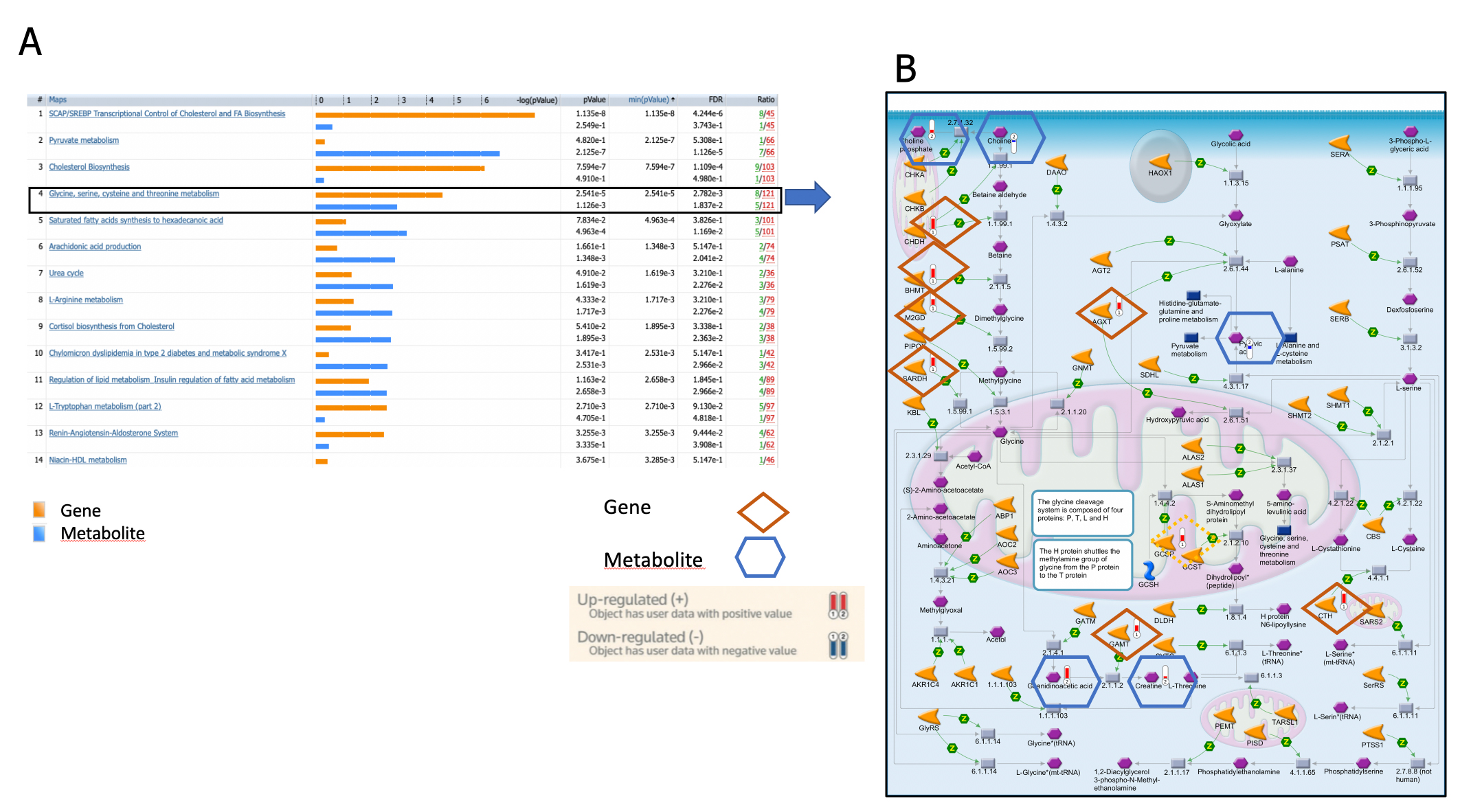


Figure S2. A) Comparison of enriched MetaCore Pathway Maps in differentially modulated genes and metabolites levels. B) Map of MetaCore Pathway Map «Glycine, serine, cysteine and threonine metabolism” in A) significantly (FDR < 0.05) enriched in both genes and metabolites differentially modulated by CPM


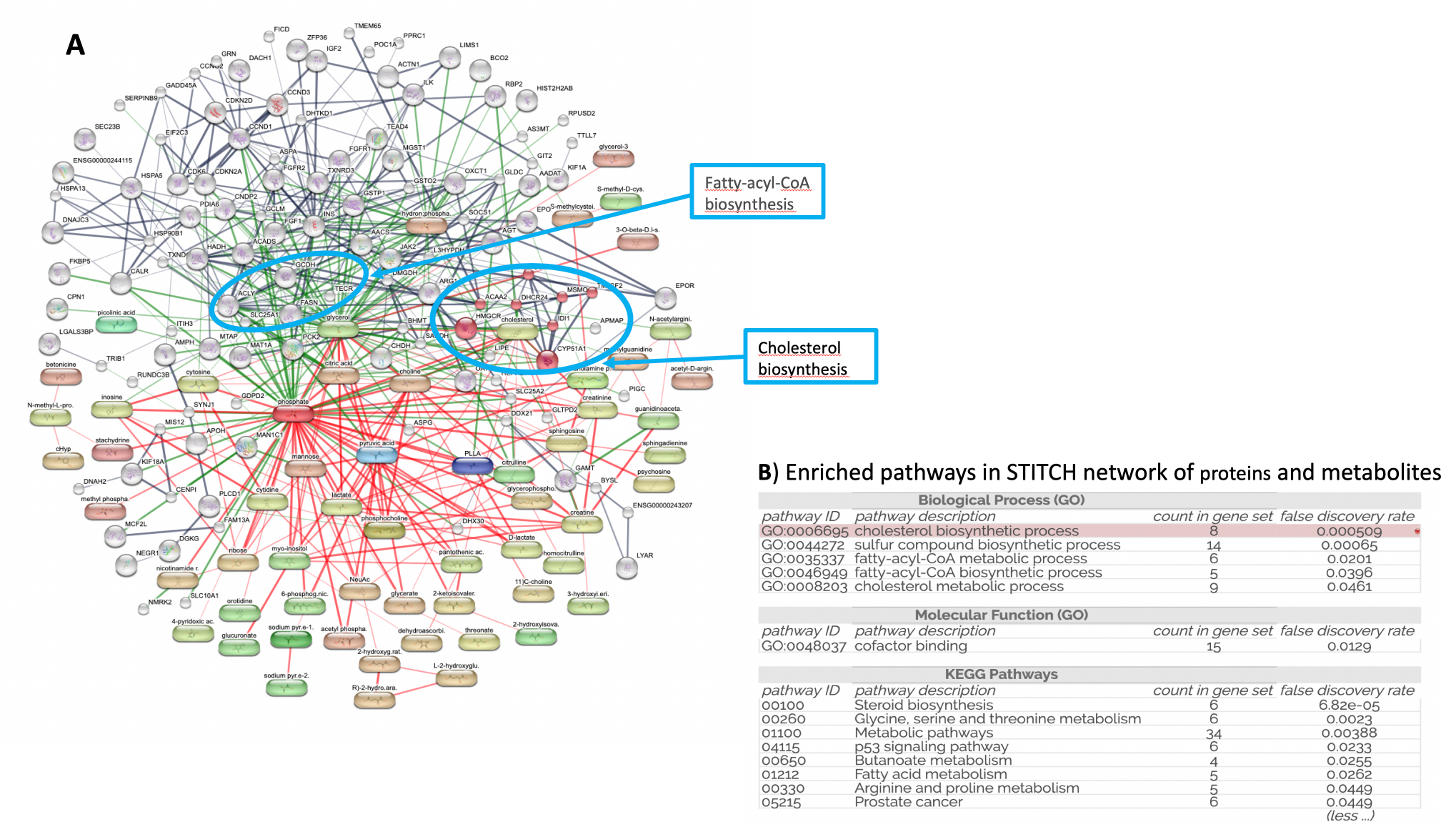


Figure S3: STITCH network of proteins and metabolites. A) Network of proteins encoding the differentially expressed genes (human orthologs) and modulated metabolites generated using the STICTH database. The manually highlighted subnetworks represent two of the top enriched pathways cholesterol and fatty-acyl-COA biosynthesis. B) Disconnected network elements were removed to enhance visualization.

in Atlantic cod. A combined list of differentially affected metabolites (p< 0.05) and genes differentially regulated (FDR q-value<0.1) by the three doses were used in pathway enrichment analysis in MetaCore.

Table S1. Summary of RNA-seq data quality.

|  |  |  |  |  |  |  |  |
| --- | --- | --- | --- | --- | --- | --- | --- |
|  |  |  |  |  |  |  |  |
| **Sample name** | **Raw reads** | **Clean reads** | **Clean bases** | **Error rate (%)** | **Q20 (%)** | **Q30 (%)** | **GC content (%)** |
|  |  |  |  |  |  |  |  |
|  |  |  |  |  |  |  |  |
| Control1 | 65576444 | 63995400 | 9.6G | 0.03 | 96.58 | 91.66 | 55.04 |
| Control2 | 56694022 | 55466278 | 8.3G | 0.03 | 95.92 | 90.23 | 55.2 |
| Control3 | 55953646 | 54723942 | 8.2G | 0.03 | 95.98 | 90.41 | 55.24 |
| Control4 | 53775298 | 52679576 | 7.9G | 0.03 | 96.64 | 91.78 | 54.4 |
| Control5 | 46376694 | 45485480 | 6.8G | 0.03 | 94.47 | 86.75 | 54.36 |
| Control6 | 49922212 | 48875480 | 7.3G | 0.03 | 95.77 | 89.95 | 54.68 |
| Control7 | 51522790 | 50286894 | 7.5G | 0.03 | 96.16 | 90.79 | 54.46 |
| Control8 | 50097642 | 49027618 | 7.4G | 0.03 | 96.19 | 90.82 | 54.9 |
| Control9 | 59175062 | 57644194 | 8.6G | 0.03 | 95.63 | 89.69 | 54.81 |
| Low1 | 40593684 | 39870928 | 6G | 0.03 | 96.55 | 91.55 | 54.46 |
| Low2 | 47173564 | 46113498 | 6.9G | 0.03 | 96.56 | 91.57 | 55.37 |
| Low3 | 54891476 | 53539560 | 8G | 0.03 | 96.16 | 90.72 | 55.56 |
| Low4 | 48811242 | 47679876 | 7.2G | 0.03 | 96.68 | 91.83 | 55.02 |
| Low5 | 49752314 | 48752566 | 7.3G | 0.03 | 96.37 | 91.17 | 54.86 |
| Low6 | 55145282 | 54025988 | 8.1G | 0.03 | 96.57 | 91.71 | 54.74 |
| Low7 | 53091940 | 52102198 | 7.8G | 0.03 | 96.67 | 91.84 | 54.83 |
| Low8 | 51448038 | 50395272 | 7.6G | 0.03 | 96.15 | 90.77 | 54.59 |
| Low9 | 46980424 | 46045010 | 6.9G | 0.03 | 96.36 | 91.2 | 54.49 |
| Medium1 | 53631780 | 52521740 | 7.9G | 0.03 | 96.26 | 90.94 | 54.48 |
| Medium2 | 54930862 | 53694802 | 8.1G | 0.03 | 96.42 | 91.41 | 55.06 |
| Medium3 | 56336298 | 55005684 | 8.3G | 0.03 | 96.02 | 90.5 | 54.58 |
| Medium4 | 50464826 | 49258072 | 7.4G | 0.03 | 96.51 | 91.51 | 55.02 |
| Medium5 | 46229698 | 45209048 | 6.8G | 0.03 | 96.69 | 91.97 | 54.45 |
| Medium6 | 52915630 | 51567724 | 7.7G | 0.03 | 96.79 | 92.18 | 55.01 |
| Medium7 | 42988646 | 41832960 | 6.3G | 0.03 | 96.19 | 90.83 | 55.18 |
| Medium8 | 48148256 | 46850922 | 7G | 0.03 | 96.61 | 91.78 | 55.46 |
| Medium9 | 49018040 | 47846546 | 7.2G | 0.03 | 96.16 | 90.81 | 54.82 |
| High1 | 42856660 | 41892404 | 6.3G | 0.03 | 96.23 | 91.02 | 55.29 |
| High2 | 54531896 | 53272770 | 8G | 0.03 | 96.42 | 91.39 | 55.76 |
| High3 | 45335296 | 44294488 | 6.6G | 0.03 | 96.76 | 92.09 | 54.81 |
| High4 | 53611394 | 52130546 | 7.8G | 0.03 | 95.86 | 90.2 | 55.22 |
| High5 | 49916788 | 48562270 | 7.3G | 0.03 | 96.35 | 91.25 | 54.88 |
| High6 | 45376706 | 44188488 | 6.6G | 0.03 | 96.54 | 91.61 | 55.19 |
| High7 | 40374966 | 39380886 | 5.9G | 0.03 | 97.23 | 93.08 | 54.56 |
| High8 | 54106310 | 52801704 | 7.9G | 0.03 | 96.93 | 92.41 | 54.85 |
| High9 | 52067772 | 50531076 | 7.6G | 0.03 | 96.21 | 90.9 | 55.58 |
|  |  |  |  |  |  |  |  |
|  |  |  |  |  |  |  |  |

Table S2. Enriched Wikipathways (Enrichr tool) using genes differentially regulated by Low, Medium and High dose groups.

|  |  |  |  |  |  |  |
| --- | --- | --- | --- | --- | --- | --- |
|  |  |  |  |  |  |  |
| **Term** | **Overlap** | **P-value** | **Adjusted P-value** | **Z-score** | **Combined Score** | **Genes** |
|  |  |  |  |  |  |  |
|  |  |  |  |  |  |  |
| **Low dose** |  |  |  |  |  |  |
| EPO Receptor Signaling WP581 | 2/26 | 2.42983E-05 | 0.000874739 | -2.578037155 | 27.39191247 | SOCS1;EPO |
| Leptin Insulin Overlap WP3935 | 1/17 | 0.00508981 | 0.032525699 | -3.218920015 | 16.99755491 | SOCS1 |
| TP53 Network WP1742 | 1/19 | 0.005687189 | 0.032525699 | -3.200698772 | 16.54613776 | GADD45A |
| Imatinib and Chronic Myeloid Leukemia WP3640 | 1/20 | 0.005985766 | 0.032525699 | -2.541995307 | 13.0108748 | GADD45A |
| The human immune response to tuberculosis WP4197 | 1/23 | 0.006881051 | 0.032525699 | -2.514968176 | 12.52198614 | SOCS1 |
| Spinal Cord Injury WP2431 | 2/118 | 0.000509794 | 0.007418982 | -1.457169444 | 11.04753612 | ZFP36;GADD45A |
| Type II interferon signaling (IFNG) WP619 | 1/37 | 0.011050164 | 0.032525699 | -2.320503793 | 10.45458898 | SOCS1 |
| ATM Signaling Pathway WP2516 | 1/40 | 0.011941645 | 0.032525699 | -2.230531128 | 9.876174872 | GADD45A |
| Differentiation Pathway WP2848 | 1/48 | 0.014315655 | 0.032525699 | -2.109546557 | 8.95798187 | EPO |
| Endometrial cancer WP4155 | 1/63 | 0.018754112 | 0.032525699 | -1.992755438 | 7.923877621 | GADD45A |
| Adipogenesis WP236 | 2/130 | 0.000618248 | 0.007418982 | -1.064850842 | 7.867778353 | SOCS1;GADD45A |
| Photodynamic therapy-induced HIF-1 survival signaling WP3614 | 1/37 | 0.011050164 | 0.032525699 | -1.71141043 | 7.710434551 | EPO |
| Kit receptor signaling pathway WP304 | 1/59 | 0.017572155 | 0.032525699 | -1.819323047 | 7.352684421 | SOCS1 |
| Oxidative Damage WP3941 | 1/40 | 0.011941645 | 0.032525699 | -1.642868189 | 7.274165924 | GADD45A |
| G1 to S cell cycle control WP45 | 1/64 | 0.019049416 | 0.032525699 | -1.835729705 | 7.270809209 | GADD45A |
| Interferon type I signaling pathways WP585 | 1/54 | 0.016093041 | 0.032525699 | -1.757077043 | 7.255618302 | SOCS1 |
| IL-4 Signaling Pathway WP395 | 1/54 | 0.016093041 | 0.032525699 | -1.752565575 | 7.236988789 | SOCS1 |
| Preimplantation Embryo WP3527 | 1/58 | 0.017276481 | 0.032525699 | -1.569362809 | 6.369116458 | ZFP36 |
| Hematopoietic Stem Cell Differentiation WP2849 | 1/55 | 0.016389012 | 0.032525699 | -1.542918482 | 6.343160298 | EPO |
| miRNA Regulation of DNA Damage Response WP1530 | 1/71 | 0.021114471 | 0.032525699 | -1.48515307 | 5.729418534 | GADD45A |
| Non-small cell lung cancer WP4255 | 1/66 | 0.019639802 | 0.032525699 | -1.426378266 | 5.605947676 | GADD45A |
| Genotoxicity pathway WP4286 | 1/63 | 0.018754112 | 0.032525699 | -1.342193668 | 5.337021375 | GADD45A |
| Viral Acute Myocarditis WP4298 | 1/84 | 0.024939962 | 0.034532255 | -1.414028248 | 5.219579649 | SOCS1 |
| Pancreatic adenocarcinoma pathway WP4263 | 1/89 | 0.026407984 | 0.035210645 | -1.348216311 | 4.899537942 | GADD45A |
| DNA Damage Response WP707 | 1/68 | 0.020229891 | 0.032525699 | -1.185520487 | 4.624234088 | GADD45A |
| Chromosomal and microsatellite instability in colorectal cancer WP4216 | 1/73 | 0.021703821 | 0.032525699 | -1.155669768 | 4.426523729 | GADD45A |
| Prolactin Signaling Pathway WP2037 | 1/76 | 0.022587291 | 0.032525699 | -1.13271086 | 4.293390853 | SOCS1 |
| Cell Cycle WP179 | 1/120 | 0.035468668 | 0.045602573 | -0.93249053 | 3.113684316 | GADD45A |
|  |  |  |  |  |  |  |
| **Medium dose** |  |  |  |  |  |  |
| Glycolysis and Gluconeogenesis WP534 | 2/45 | 0.000916601 | 0.020783363 | -1.926464818 | 13.47531071 | MDH1;GOT2 |
| One carbon metabolism and related pathways WP3940 | 2/52 | 0.001222551 | 0.020783363 | -1.616504961 | 10.84160106 | DMGDH;SARDH |
| Amino Acid metabolism WP3925 | 2/91 | 0.003688396 | 0.035518692 | -1.409167722 | 7.894951761 | MDH1;GOT2 |
| Human Complement System WP2806 | 2/97 | 0.00417867 | 0.035518692 | -0.937750809 | 5.136776085 | C3;GNAI2 |
|  |  |  |  |  |  |  |
| **High dose** |  |  |  |  |  |  |
| Cholesterol Biosynthesis Pathway WP197 | 4/15 | 2.82777E-07 | 2.37533E-05 | -3.28660533 | 49.55742732 | SQLE;CYP51A1;MSMO1;HMGCR |
| Sterol Regulatory Element-Binding Proteins (SREBP) signalling WP1982 | 5/69 | 7.32652E-06 | 0.000307714 | -1.592362926 | 18.82811557 | MBTPS1;ACLY;SQLE;CYP51A1;HMGCR |
| Tryptophan metabolism WP465 | 3/46 | 0.000767728 | 0.021496394 | -2.237236276 | 16.04562541 | OGDH;DHCR24;CYP7B1 |
| TCA Cycle and Deficiency of Pyruvate Dehydrogenase complex (PDHc) WP2453 | 2/16 | 0.001739132 | 0.02932352 | -2.966415742 | 18.84970144 | ACLY;OGDH |
| Oxidation by Cytochrome P450 WP43 | 3/61 | 0.001745448 | 0.02932352 | -1.37619553 | 8.73986582 | CYP2U1;CYP51A1;CYP7B1 |
| Fatty Acid Biosynthesis WP357 | 2/22 | 0.003297491 | 0.046164874 | -2.340160154 | 13.37306378 | ACLY;ACAA2 |
| Statin Pathway WP430 | 2/29 | 0.00569424 | 0.048009911 | -2.675285292 | 13.82667718 | SQLE;HMGCR |
| Lipid Metabolism Pathway WP3965 | 2/29 | 0.00569424 | 0.048009911 | -2.131849644 | 11.01803868 | LIPE;ACLY |
| Amino Acid metabolism WP3925 | 3/91 | 0.005419463 | 0.048009911 | -1.373460951 | 7.166387524 | ACLY;MARS2;OGDH |
| Metapathway biotransformation Phase I and II WP702 | 4/183 | 0.005715466 | 0.048009911 | -0.963147992 | 4.974254381 | CYP2U1;GSTO2;CYP51A1;CYP7B1 |
|  |  |  |  |  |  |  |
|  |  |  |  |  |  |  |

Table S3. Pathways enriched by both differentially expressed genes and altered metabolites as determined with the IMPaLA integrated transcriptomics and metabolomics pathway analysis tool.

|  |  |  |  |  |  |  |  |  |  |  |  |  |  |
| --- | --- | --- | --- | --- | --- | --- | --- | --- | --- | --- | --- | --- | --- |
|  |  |  |  |  |  |  |  |  |  |  |  |  |  |
| **Pathway name** | **Pathway source** | **Number of measured genes** | **Measured genes** | **Number of all pathway genes** | **P-genes** | **Q-genes** | **Number of measured metabolites** | **Measured metabolites** | **Number of all pathway metabolites** | **P-metabolites** | **Q-metabolites** | **P-joint** | **Q-joint** |
|  |  |  |  |  |  |  |  |  |  |  |  |  |  |
|  |  |  |  |  |  |  |  |  |  |  |  |  |  |
| Metabolism | Reactome | 56 | AS3MT;GSTP1;FAH;MAT1A;FASN;LIPE;CNDP2;SLC10A1;ACLY;MGST1;ACADS;DMGDH;SPR;GCLM;GLDC;SLC25A2;SCD;SLC25A1;TECR;CHDH;DHCR24;CYP24A1;SQLE;EBP;CYP51A1;RBP2;AACS;SRM;TM7SF2;THEM5;GAMT;AADAT;CYP2U1;OXCT1;OAT;GCDH;ACAA2;BCO2;HMGCR;PLCD1;HADH;SYNJ1;SARDH;IDI1;GSTO2;DCK;LSS;ASPA;MSMO1;DHTKD1;AK7;CTH;ASPG;NMRK2;BHMT;ARG1 | 1972 | 9.1e-09 | 4.18e-05 | 41 | C00158;C00159;C00116;C00074;C00249;C00794;C00170;C03150;C00588;C00581;C00187;C00186;C16537;C00009;C16513;C00270;C00319;C01885;C00022;C06428;C00354;C00791;C00141;C00258;C00670;C00294;C00847;C00864;C01595;C00475;C05382;C00121;C00191;C00114;C00137;C00059;C00300;C00327;C00345;C01233;C00346 | 1384 | 8.62e-05 | 368 | 2.27e-11 | 1.12e-08 |
| Metabolism of lipids | Reactome | 25 | OXCT1;FASN;LIPE;SLC10A1;ACLY;ACADS;SCD;SLC25A1;TECR;SYNJ1;DHCR24;CYP24A1;SQLE;EBP;CYP51A1;AACS;TM7SF2;THEM5;CYP2U1;LSS;ACAA2;MSMO1;HMGCR;HADH;IDI1 | 664 | 1.23e-05 | 0.0189 | 18 | C00114;C00158;C00116;C00009;C00187;C00249;C00670;C01233;C00588;C16513;C00270;C00059;C01595;C00319;C01885;C00137;C06428;C00346 | 620 | 0.0268 | 1 | 5.27e-06 | 0.0013 |
| Metabolism of amino acids and derivatives | Reactome | 18 | GAMT;SARDH;SRM;DHTKD1;DMGDH;FAH;ASPG;GLDC;SLC25A2;OAT;MAT1A;CTH;CHDH;GCDH;BHMT;ASPA;AADAT;ARG1 | 342 | 7.63e-06 | 0.0175 | 10 | C00114;C00009;C00022;C00791;C00170;C00059;C00300;C00327;C00581;C00141 | 285 | 557 | 1 | 5.68e-05 | 0.00935 |
| Fatty acid metabolism | Reactome | 10 | ACADS;THEM5;CYP2U1;SCD;SLC25A1;ACAA2;FASN;TECR;HADH;ACLY | 189 | 0.00195 | 578 | 6 | C00158;C00009;C00249;C16513;C01595;C06428 | 367 | 0.0313 | 1 | 0.000653 | 0.0807 |
|  |  |  |  |  |  |  |  |  |  |  |  |  |  |
|  |  |  |  |  |  |  |  |  |  |  |  |  |  |
